# Establishment and Characterization of a New Cell Line Permissive for Hepatitis C Virus Infection

**DOI:** 10.1038/s41598-019-44257-5

**Published:** 2019-05-28

**Authors:** Hitoshi Omura, Fanwei Liu, Tetsuro Shimakami, Kazuhisa Murai, Takayoshi Shirasaki, Juria Kitabayashi, Masaya Funaki, Tomoki Nishikawa, Ryotaro Nakai, Ariunaa Sumiyadorj, Takehiro Hayashi, Taro Yamashita, Masao Honda, Shuichi Kaneko

**Affiliations:** 1 Department of Gastroenterology, Kanazawa University Graduate School of Medicine, Kanazawa, Ishikawa 920-8641 Japan; 2 State Key Laboratory for Diagnosis and Treatment of Infectious Diseases, Collaborative Innovation Center for Diagnosis and Treatment of Infectious Diseases, The First Affiliated Hospital, College of Medicine, Zhejiang University, Hangzhou, 310003 China

**Keywords:** Hepatitis C virus, Hepatitis C

## Abstract

Hepatitis C virus (HCV) cell culture systems have facilitated the development of efficient direct-acting antivirals against HCV. Huh-7.5, a subline of the human hepatoma cell line Huh-7, has been used widely to amplify HCV because HCV can efficiently replicate in these cells due to a defect in innate antiviral signalling. Recently, we established a novel cell line, KH, derived from human hepatocellular carcinoma, which showed atypical uptake of gadolinium ethoxybenzyl diethylenetriamine pentaacetic acid (Gd-EOB-DTPA) in a Gd-EOB-DTPA-enhanced magnetic resonance imaging study. KH cells expressed hepatocyte markers including microRNA-122 (miR-122) at a lower level than Huh-7.5 cells. We demonstrated that KH cells could support the entire life cycle of HCV; however, HCV replicated at a lower rate in KH cells compared to Huh-7.5 cells, and virus particles produced from KH cells seemed to have some disadvantages in viral assembly compared with those produced from Huh-7.5 cells. KH cells had more robust interferon-stimulated gene expression and induction upon HCV RNA transfection, interferon-α2b addition, and HCV infection than Huh-7.5 cells. Interestingly, both miR-122 supplementation and IRF3 knockout in KH cells boosted HCV replication to a similar level as in Huh-7.5 cells, suggesting that intact innate antiviral signalling and lower miR-122 expression limit HCV replication in KH cells. KH cells will enable a deeper understanding of the role of the innate immune response in persistent HCV infection.

## Introduction

Hepatitis C virus (HCV) is a positive-stranded RNA virus that causes acute infection and a subsequent persistent infection in 70–80% of infected individuals. Persistent HCV infection in the liver can cause chronic hepatitis, liver cirrhosis, and/or hepatocellular carcinoma (HCC)^1^. At present, approximately 180 million people worldwide are infected with HCV^2^ and ~704,000 people die of HCV-related liver disease each year^3^. Therefore, antiviral treatments that can eradicate HCV completely from the liver, a status defined as a sustained viral response (SVR), are desperately needed to reduce the number of deaths from liver disease.

The establishment of HCV cell culture systems has facilitated the development of direct-acting antivirals (DAAs). Current antiviral treatments against HCV involve the use of a combination of DAAs without the use of interferon (IFN), that is, IFN-free DAA treatment. The latest IFN-free DAA treatment is reported to eliminate HCV in more than 95% of patients and has fewer adverse effects and a shorter treatment period compared with IFN-based treatments^4^. However, treatment failure due to resistance-associated variants or viral relapse must be overcome to achieve a 100% SVR.

The endogenous IFN response is activated continuously by HCV in all patients during acute and chronic infection; however, it is not sufficiently effective to eradicate HCV once a persistent infection is established. More robust activation of endogenous IFN systems in the liver, determined by mRNA expression of IFN-stimulated genes (ISGs), is associated with treatment failure in IFN-based antiviral therapies; patients with higher ISG levels have an inferior response to IFN-based therapy and significantly lower SVR rates than those with lower ISG levels^5^. These results indicate that intrahepatic ISGs or endogenous IFN systems play an important role in antiviral defence against HCV. Although the impact of ISG levels on treatment outcomes has been attenuated in the era of IFN-free DAA treatment, one report has shown that intrahepatic IFN signalling or ISGs may facilitate HCV eradication and prevent relapse upon DAA withdrawal^6^, suggesting that the intrahepatic innate immune response plays an important role in the eradication of HCV.

Huh-7.5 cells^7^ are the most commonly used cell line to amplify HCV because the virus can replicate efficiently due to a defect in the innate antiviral signalling pathway of these cells^8^. Although this cell line is useful for the development or screening of antiviral agents, it does not completely mimic the interactions between host innate antiviral responses and HCV. Therefore, a cell line possessing an intact innate antiviral response is needed to enable a deeper understanding of the mechanisms underlying HCV evasion of the host innate antiviral response and the establishment of a persistent infection. Recently, we established a novel cell line, namely KH, derived from human HCC, which showed atypical uptake of gadolinium ethoxybenzyl diethylenetriamine pentaacetic acid (Gd-EOB-DTPA) in a dynamic magnetic resonance imaging (MRI) study with Gd-EOB-DTPA injection^9^. In the present study, we demonstrated that KH cells could support the entire HCV life cycle. In addition, KH cells showed lower microRNA-122 (miR-122) expression and more robust ISG expression upon HCV RNA transfection and infection than Huh-7.5 cells, both of which limited HCV replication in KH cells. This cell line will facilitate a deeper understanding of the mechanisms underlying persistent HCV infection in the face of the innate immune defence system.

## Results

We used the KH cell line derived from human HCC, as described in our previous work^9^. Briefly, primary HCC tissues were obtained from patients by surgical resection. After CD45+ leukocytes and annexin V1 apoptotic cells were removed by an autoMACS Pro Separator and magnetic beads, the cells were transplanted subcutaneously into NOD/SCID mice, and the subcutaneous tumours that grew were dissected and digested to establish the cell line. Typical HCC loses the ability to uptake Gd-EOB-DTPA, which can be revealed by a dynamic Gd-EOB-DTPA-enhanced MRI study, due to the lack of its specific transporter, OATP1B; however, the original human HCC was unique in terms of EOB uptake, and this feature remained in mouse tumours after subcutaneous injection of the original HCC cells^9^. This cell line, derived from EOB uptake-positive HCC, was designated KH. The Huh-7.5 cell line is a well-known and widely used system that can efficiently support the entire HCV life cycle; thus, we compared KH and Huh-7.5 cells in the present study. The patient, whose HCC was resected, was infected with genotype 1b HCV; however, HCV RNA was undetectable in KH cells by quantitative RT-PCR (qRT-PCR). The single nucleotide polymorphism at rs8099917 related to the IFNL3 and IFNL4 variants^10^ in this patient and t KH cell line was an unfavourable non-TT variant (data not shown).

### Multicolor fluorescent *in situ* hybridization (FISH) analysis of chromosomes

**Figure 1:**
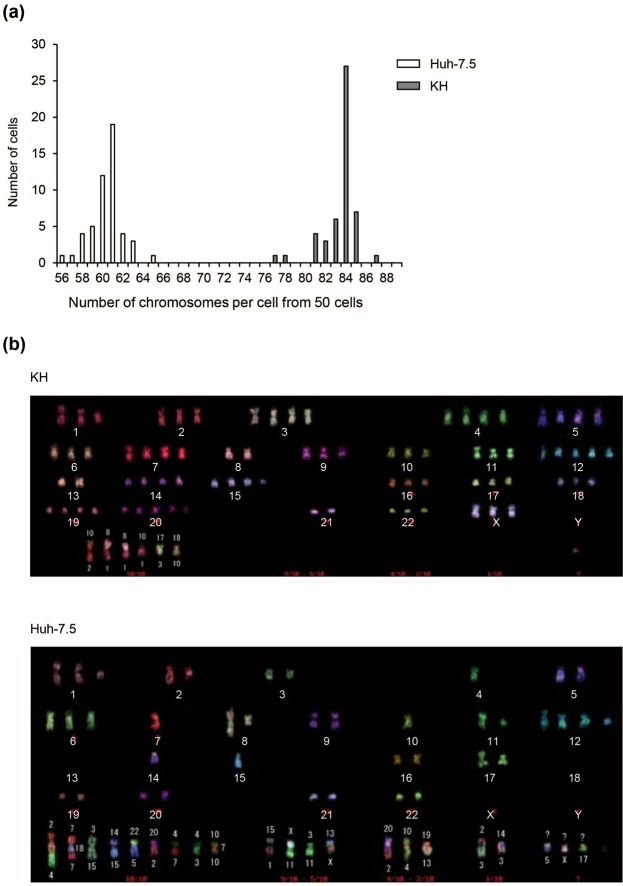
Chromosome pattern analysis of KH and Huh-7.5 cells. (**a**) Number of chromosomes in KH and Huh-7.5 cells. After metaphase, chromosome spreads were prepared from both cell lines and multicolor-FISH chromosomal analysis was performed. The number of chromosomes from 50 cells is shown. (**b**) Representative chromosome patterns in KH and Huh-7.5 cells. A representative chromosome pattern from multicolor-FISH analysis is shown. Each number or character denotes the nomenclature of each chromosome.

We performed multicolor FISH analysis of the chromosomes of KH and Huh-7.5 cells. The chromosomes were probed and analysed after metaphase chromosome spreads from both cell lines were prepared. The number of chromosomes from 50 cells ranged from 56 to 65 and peaked at 61 per cell among Huh-7.5 cells, and 77 to 87 and a peak of 84 per cell were observed among KH cells (Fig. 1a). A report showed that the number of chromosomes in Huh-7.5 cells is between 55 and 63, which is consistent with our results^11^. We also observed several types of chromosomal abnormalities including translocations, deletions, and duplications in both cell types. While translocations between chromosome 2 and 4, t(2;4), t(7;18;7), t(3;15), t(14;15), t(22;5), t(20;2), t(4;7), t(4;3), and t(10;7;10) were observed in all Huh-7.5 cells examined, t(10;2), t(8;1), t(10,1), t(17;3), and t(18;10) were observed in all KH cells examined, the translocation patterns were distinct between KH and Huh-7.5 cells (Fig. 1b). These results are compatible with the fact that both cell types are derived from hepatoma and show that both cells have distinct chromosomal patterns.

### Expression of hepatocyte, stem cell, and hepatoma markers in KH cells

**Figure 2:**
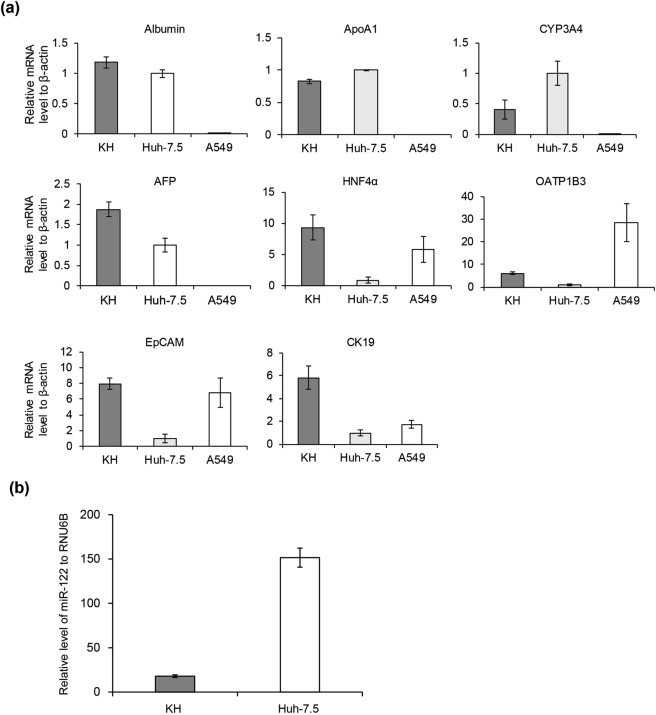
mRNA levels of hepatocyte, stem cell, and hepatoma markers and miR-122 level. (**a**) The mRNA levels of albumin, ApoA1, CYP3A4, HNF4α, AFP, OATP1B3, CK19, and EpCAM, in KH, Huh-7.5, and A549 cells were determined using qRT-PCR. (**b**) The RNA levels of miR-122 and RNU6B were quantified in KH and Huh-7.5 cells, and the level of miR-122 was normalised to that of RNU6B.

We examined the mRNA expression levels of several hepatocyte/hepatoma markers, such as albumin, ApoA1, CYP3A4, HNF4α, OATP1B3, and AFP in both cell lines and in lung carcinoma-derived A549 cells by qRT-PCR (Fig. 2a). mRNAs of hepatocyte/hepatoma markers, such as albumin, ApoA1, CYP3A4, and AFP, were detectable in KH and Huh-7.5 cells, but not in A549 cells, indicating that KH cells, similar to Huh-7.5 cells, are derived from hepatocyte/hepatoma cells. mRNAs of HNF4α and OATP1B3 were detected in KH, Huh-7.5, and A549 cells, which is compatible with the reports that OATP1B and HNF4α can be expressed in lung cancer^12,13^. We also examined the mRNA levels of the stem cell markers CK19 and EpCAM. Interestingly, CK19 and EpCAM mRNA levels were much higher in KH cells than in Huh-7.5 cells.

### miR-122 abundance in KH cells

As a liver-specific microRNA (miRNA), miR-122 is an important host factor for HCV replication^14^. Therefore, we compared the abundance of miR-122 in KH and Huh-7.5 cells. miR-122 was expressed at a lower level in KH cells than in Huh-7.5 cells; Huh-7.5 cells contained approximately 8.4-fold more miR-122 than KH cells (Fig. 2b). This result suggests that the lower level of miR-122 in KH cells could partially explain the lower capacity of KH cells to support HCV replication compared to Huh-7.5 cells, as presented elsewhere, and this possibility is addressed later in this work.

### Efficient replication of HCV in KH cells

To examine whether KH cells could support HCV replication, we transfected an *in vitro* transcribed HCV RNA of HJ3-5, which is a chimeric clone of genotype 1a H77S in the structural region and genotype 2a JFH1 in the nonstructural region, into KH and Huh-7.5 cells. HJ3-5/GND is a replication-defective mutant of HJ3-5 because the catalytic centre motif of NS5B (GDD) is mutated to GND.

**Figure 3:**
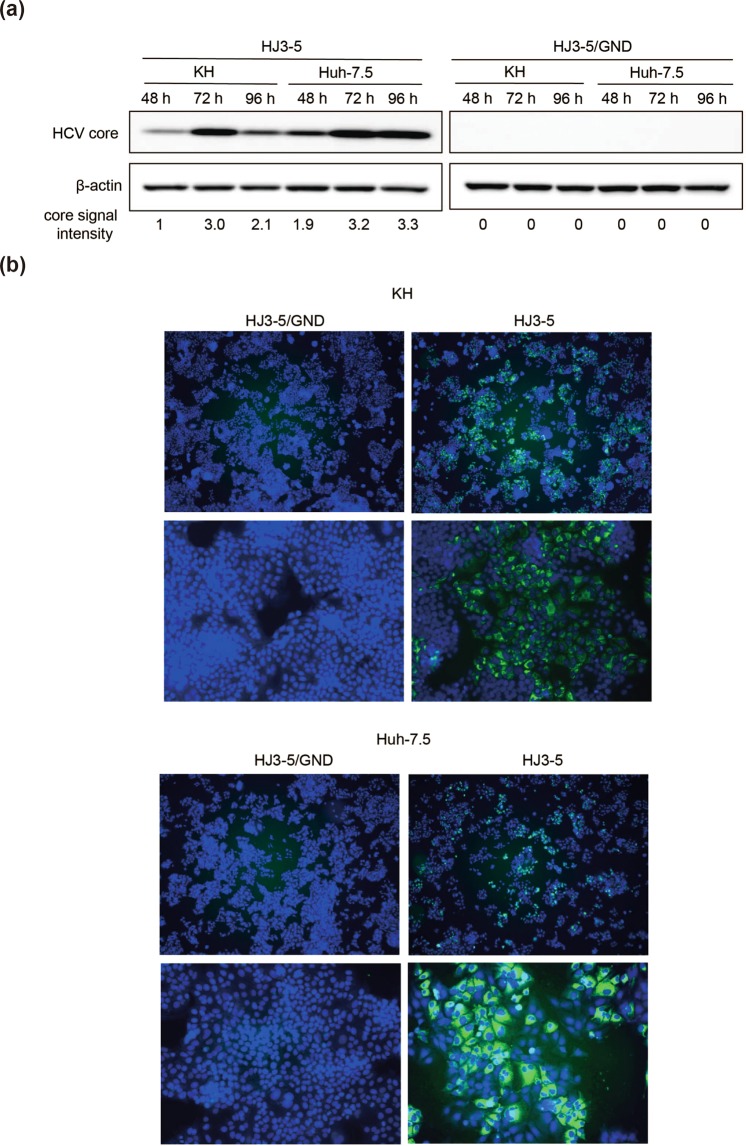
Efficient replication of HCV in KH cells. HCV RNA of HJ3-5, a chimeric clone of genotype 1a H77S and genotype 2a JFH-1, and HJ3-5/GND, a replication-defective NS5B mutant of HJ3-5, were transfected by electroporation into KH and Huh-7.5 cells. (**a**) HCV core protein and β-actin expression by western blotting analysis with appropriate antibodies at 48, 72, and 96 h after transfection. Signal strength of HCV core protein and β-actin was quantitated by ImageJ, normalised to β-actin at each time point, and further normalised to the signal strength of HCV core protein of KH cells at 48 h, which was set to 1. Full-length gels and blots before cropping are shown in Supplementary Fig. S3. (**b**) Immunofluorescence analysis of HCV core protein expression at 96 h after transfection. Nuclei are labelled with DAPI. Images are merged; each image was taken under the same conditions. Upper panels, low power field; lower panels, high power field. Blue, DAPI; green, HCV core.

When we assessed the expression of HCV core protein using western blotting and an immunofluorescence assay with an anti-core antibody, we observed its expression in KH and Huh-7.5 cells (Fig. 3a,b). When we compared HCV core protein expression level between both cell lines, the level of HCV core protein expression in Huh-7.5 cells was 1.9, 1.1, and 1.6 times higher than in KH cells at 48, 72, and 96 h after transfection, respectively. Conversely, HCV core protein expression was not observed in HJ3-5/GND RNA-transfected cells. These results suggested that KH cells have the ability to support HCV replication, similar to Huh-7.5 cells.

### Replication of several HCV genotypes in KH cells

**Figure 4:**
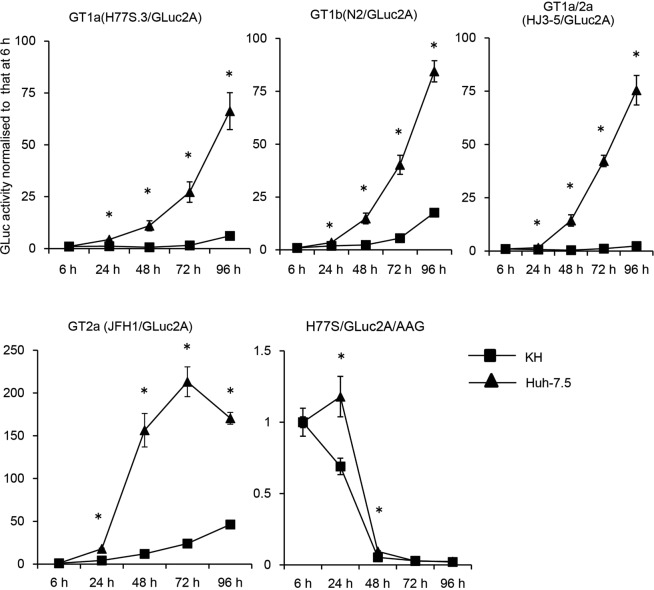
Replication of several HCV genotypes. H77S.3/GLuc2A (genotype 1a), HJ3-5/GLuc2A (genotype 1a/2a chimaera), N2/GLuc2A (genotype 1b), and JFH1/GLuc2A (genotype 2a) RNA were transfected into KH and Huh-7.5 cells. A replication-deficient mutant, H77S/GLuc2A/AAG, was also transfected. Medium was replaced at 6 h and then at 24 h intervals until 96 h. Secreted GLuc activity was determined and normalised to that at 6 h. GT denotes genotype. Differences in the means of normalised GLuc activity between KH and Huh-7.5 cells at each time point were analyzed by Student’s t test.

We further examined whether transfected HCV RNAs from genotypes other than the 1a/2a chimaera, namely, 1a H77S.3, 1b N2, and 2a JFH1, could replicate in KH cells. For this purpose, we used a *Gaussia* luciferase (GLuc)-containing genome inserted between p7 and NS2^15^. H77S.3/GLuc2A^15^ (genotype 1a), N2/GLuc2A^16^ (genotype 1b), JFH1/GLuc2A^16^ (genotype 2a), and HJ3-5/GLuc2A^17^ RNAs were transfected into KH and Huh-7.5 cells. We also transfected H77S/GLuc2A/AAG in which the catalytic centre of NS5B, that is, GDD, was mutated to AAG; this RNA cannot replicate due to the lack of the RNA-dependent RNA polymerase activity of NS5B. After transfection of the HCV RNAs, GLuc activity increased continuously for 72–96 h in KH and Huh-7.5 cells. In the case of H77S/GLuc2A/AAG RNA-transfected cells, GLuc activity decreased quickly to baseline by 48 h after transfection, suggesting that the GLuc activity in other HCV-RNA-transfected cells reflected efficient HCV replication. GLuc activity was significantly lower in KH cells than in Huh-7.5 cells among all HCV genotypes tested (Fig. 4). These data suggested that KH cells can support several genotypes of HCV; however, the ability of KH cells to support HCV replication is inferior to that of Huh-7.5 cells.

### Infectious virus production from KH cells and HCV entry into KH cells

**Figure 5:**
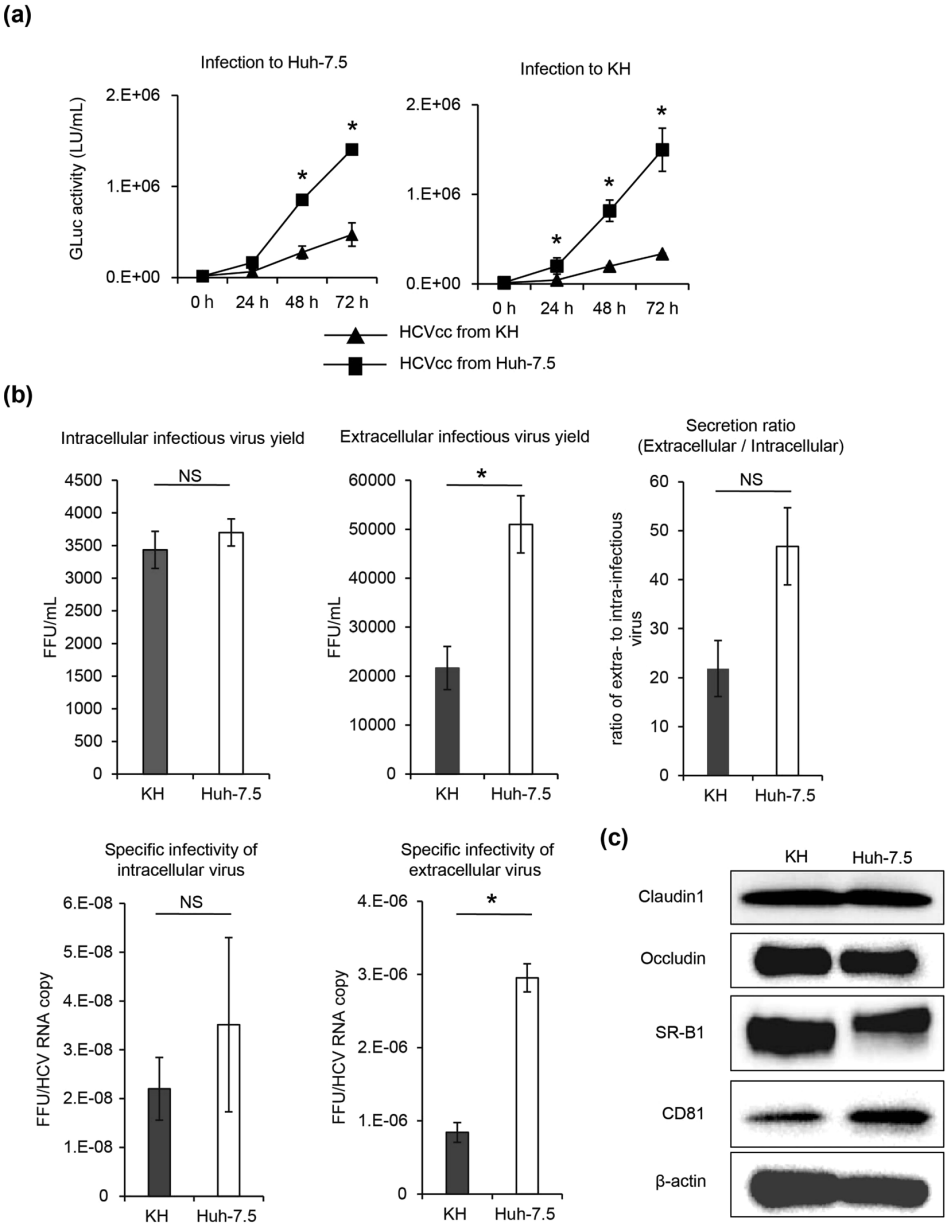
Infectious virus production from KH cells and HCV entry into KH cells. (**a**) GLuc activity analysis. HJ3-5/GLuc2A RNA was transfected into KH and Huh-7.5 cells, and at 72 h after transfection, medium was collected and used to infect both cell lines. The infected cells were inoculated, the medium was replaced every 24 h, and GLuc activity was determined every 24 h from 0–72 h after infection; the data were analysed with Student’s t test. (**b**) HJ3-5 RNA was transfected into KH and Huh-7.5 cells, and at 72 h later, intra- and extra-cellular infectious virus yield, infectious virus release from cells to the medium, and the specific infectivity of intra- and extra-cellular virus were examined. Infectious virus yield was evaluated using a conventional FFU assay, and infectious virus release was calculated by the ratio of extra- to intra-cellular infectious yield. The difference between KH and Huh-7.5 cells was analysed by Student’s t test. (**c**) Expression of representative proteins essential for HCV entry: claudin1, occludin, SR-B1, and CD81, and β-actin (western blotting). Full-length gels and blots before cropping are shown in Supplementary Fig. S4. HCVcc: cell culture-derived HCV; FFU: Focus-forming unit per mL.

We compared the production of infectious virus and permissiveness for viral entry between KH and Huh-7.5 cells. For this purpose, at first, we transfected HJ3-5/GLuc2A RNA into KH and Huh-7.5 cells, collected the media, and used them to infect KH and Huh-7.5 cells. Infection and subsequent viral replication in each cell was analysed with a GLuc assay. Huh-7.5 cells infected with media collected from KH and Huh-7.5 cells showed increased GLuc activity, and KH cells infected with media collected from KH and Huh-7.5 cells also showed increased GLuc activity (Fig. 5a). As Huh-7.5 cells are known to support the entire HCV lifecycle, such as infectious virus production in the cells, release of virus to other cells, and entry of virus into cells, successful cell culture-derived HCV (HCVcc) transfer from KH cells to Huh-7.5 cells shows that KH cells have the ability to produce and release infectious virus. Moreover, successful HCVcc transfer from Huh-7.5 cells to KH cells shows that KH cells have the ability to permit entry of HCVcc. Generally, KH and Huh-7.5 cells infected with medium collected from KH cells showed lower GLuc activity than the cells infected with medium collected from Huh-7.5 cells. Next, we compared intra- and extra-cellular infectious virus yields, infectious virus release, and specific infectivity of intra- and extra-cellular virus between KH and Huh-7.5 cells. While intracellular infectious virus yield was comparable between KH and Huh-7.5 cells, the extracellular infectious virus yield of KH cells was statistically lower than that of Huh-7.5 cells. Infectious virus release by KH cells, which was calculated using the secretion ratio of infectious virus from intra- to extra-cells, was lower than that by Huh-7.5 cells, although this difference was not statistically significant. While the specific infectivity of intracellular virus was comparable between KH and Huh-7.5 cells, the specific infectivity of extracellular virus of KH cells was statistically lower than that of Huh-7.5 cells (Fig. 5b). These results suggest that KH cells have an inferior ability in the late steps of the viral life cycle compared to Huh-7.5 cells that could involve the efficiency of viral assembly.

### Expression of essential factors for virus entry

We examined the expression of representative factors essential for virus entry. Several host proteins have been reported to play important roles in virus entry into hepatocytes; thus, we examined the expression of four of them (CD81, SR-B1, claudin1, and occludin) in KH and Huh-7.5 cells with western blot analysis and detected the substantial expression of all of these proteins in both cell lines; their expression levels seemed to be identical between KH and Huh-7.5 cells (Fig. 5c). The expression of these proteins explains the ability of HCV to enter KH cells.

### Sequence analysis of RIG-I

**Figure 6:**
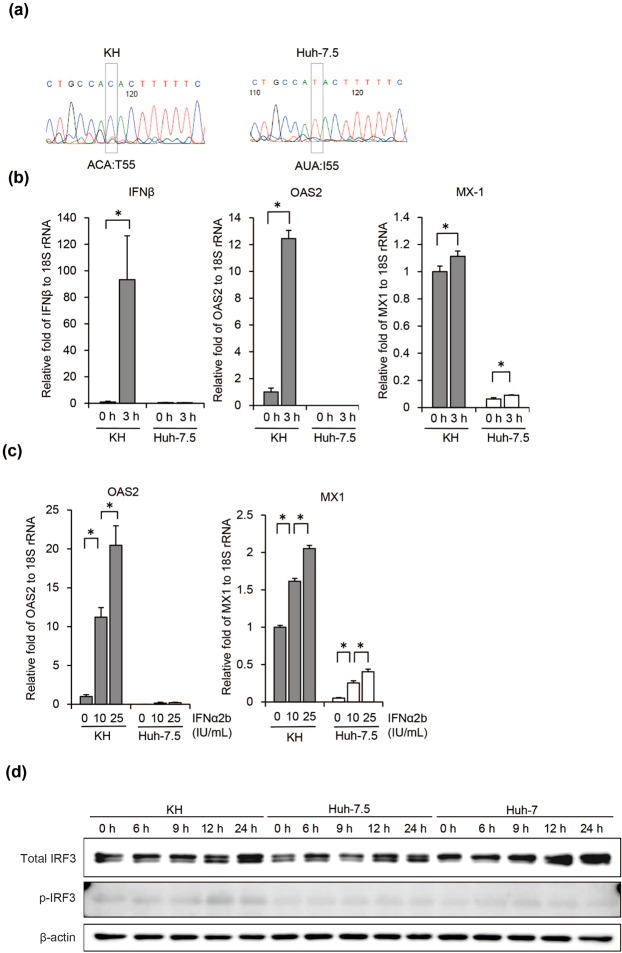
Induction of ISGs by HCV RNA, IFNα2b, and HCV infection. (**a**) RIG-I sequence analysis. The amino acid at position 55 of RIG-I from KH and Huh-7.5 cells was determined. (**b**) Effect of HCV RNA transfection on ISG mRNA levels. qRT-PCR of the ISGs IFNβ, OAS2, and MX1 mRNA, and 18S rRNA; ISG mRNA levels were normalised to 18S rRNA, and then further normalised to KH mRNA levels at 0 h, which were set to 1. Differences in mRNA levels were analysed with Student’s t test. (**c**) Effects of IFNα2b on ISG mRNA levels. KH and Huh-7.5 cells treated with 0, 10, and 25 IU/mL IFNα2b, for 3 h. qRT-PCR of OAS2 and MX1 mRNA, and 18S rRNA. ISG mRNA levels were normalised to 18S rRNA, and then further normalised to KH mRNA level at 0 IU/mL IFN IFNα2b, which was set to 1. Differences in mean relative mRNA levels were analysed with one-way analysis of variance and Tukey’s multiple comparisons test. (**d**) Effect of HCV RNA transfection on IRF3 and phosphorylated (p)-IRF3. JFH1 A338U RNA was transfected into KH, Huh-7, and Huh-7.5 cells, and IRF3, p-IRF3, and β-actin expression was analysed by western blotting with appropriate antibodies at 0, 6, 9, 12, and 24 h after transfection. Full-length gels and blots before cropping are shown in Supplementary Fig. S5.

Huh-7.5 cells are characterised by a defective retinoic-acid-inducible gene-I (RIG-I) pathway, which plays an important role in the antiviral immune response; this defect is reported to be caused by a mutation, T55I, in RIG-I^8^. The lower ability of KH cells to support HCV replication compared with Huh-7.5 cells suggested that KH cells could have an intact antiviral immune response. To test this hypothesis, we first determined the amino acid sequence at position 55 of RIG-I. As shown in a previous study, this amino acid threonine was substituted with isoleucine in Huh-7.5 cells, while in KH cells, it was threonine, which is the wild-type residue (Fig. 6a). This suggests that the RIG-I pathway could be intact in KH cells.

### Robust ISG induction in KH cells by HCV RNA transfection

Next, we examined whether the IFN signalling downstream of RIG-I was also preserved in KH cells. For this purpose, we measured the mRNA levels of some representative ISGs, namely, IFNβ, OAS2, and MX1, in KH and Huh-7.5 cells upon HCV RNA transfection. We transfected replication-deficient H77S/AAG RNA into KH and Huh-7.5 cells, extracted total RNA at 3 h after HCV RNA transfection, and quantified the mRNA levels of the ISGs normalised to the basal mRNA level. While HCV RNA failed to induce IFNβ and OAS2 expression in Huh-7.5 cells (Fig. 6b), these ISGs were dramatically induced in KH cells. The induction of MX1 expression by HCV RNA transfection was similar in KH and Huh-7.5 cells; however, its basal level was much higher in KH cells than in Huh-7.5 cells. This result shows that HCV RNA transfection induced ISG expression more efficiently in KH cells than in Huh-7.5 cells.

### Robust ISG induction in KH cells by IFNα2b

We next evaluated the mRNA levels of ISGs, such as OAS2 and MX1, in KH and Huh-7.5 cells upon the addition of IFNα2b. The cells were treated with several concentrations of IFNα2b (0, 10, and 25 IU/mL) and total RNA was extracted at 3 h later. The expression levels of OAS2 and MX1 were quantified by qRT-PCR (Fig. 6c). We found that IFNα2b induced OAS2 and MX1 expression in a dose-dependent manner in KH cells. IFNα2b failed to induce OAS2 mRNA in Huh-7.5, and although it induced MX1 expression in a dose-dependent manner in Huh-7.5 cells, its mRNA levels were much lower than in KH cells. These results indicated that ISGs were induced more efficiently by IFNα2b in KH cells than in Huh-7.5 cells.

To compare the ISG protein expression levels between KH, Huh-7.5, and Huh-7 cells, we treated these cells with HCV RNA, polyinosinic-polycytidylic acid sodium salt, poly(I:C), and IFNα2b, and performed western blot analysis for the following ISGs: MDA5, RIG-I, MAVS, IRF7, phosphorylated (p)-IRF7, IRF3, p-IRF3, IRF9, STAT1, p-STAT1, STAT2, and p-STAT2. In this experiment, we used translation-incompetent mutant HCV RNA, JFH1 A338U, with a critical mutation within the HCV internal ribosome entry sites^18^ to avoid any possible effects of viral proteins on ISG expression. While HCV RNA transfection did not greatly increase the expression of total IRF3 in these cells, it increased the phosphorylated form of IRF3 in KH cells compared to Huh-7.5 and Huh-7 cells (Fig. 6d). As IRF3 phosphorylation is known to induce IFNβ expression, the more robust induction of IFNβ mRNA expression following HCV RNA transfection in KH cells (Fig. 6b) can be explained the greater increase in the phosphorylated form of IRF3 in these cells. Interestingly, HCV RNA, poly(I:C), and IFNα2b induced the expression of IRF9 protein in Huh-7 cells, but not in KH and Huh-7.5 cells (Supplementary Fig. S1). Regarding ISGs other than those mentioned above, we did not observe significant differences in their protein expression patterns among KH, Huh-7.5, and Huh-7 cells (Supplementary Fig. S1).

### Robust ISG induction in KH cells upon HCV infection

**Figure 7:**
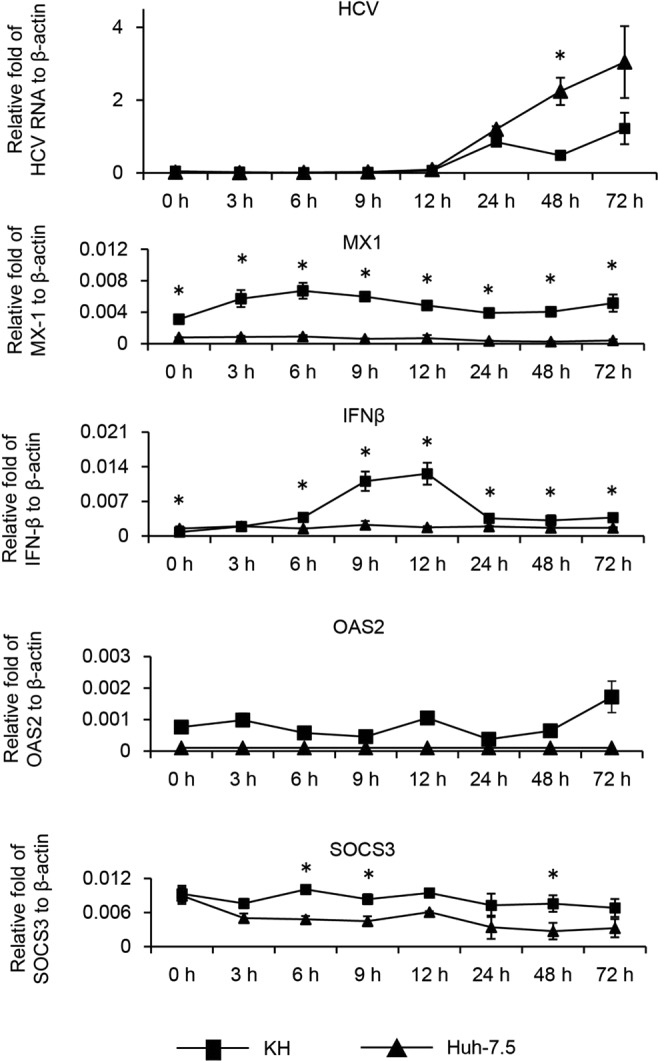
Effect of HCV infection on ISG mRNA levels. HCVcc derived from HJ3-5 was used to infect Huh-7.5 and KH cells at a multiplicity of infection of 1. Total cellular RNA was extracted at regular intervals after infection, and HCV RNA and mRNA levels of ISGs, such as MX1, SOCS3, IFNβ, and OAS2, together with β-actin for normalisation were quantified using qRT-PCR. Differences in the means of mRNA levels between KH and Huh-7.5 cells at each time point were analysed with Student’s t test.

We also measured ISG levels following HCV infection. HCVcc derived from HJ3-5 virus was used to infect Huh-7.5 and KH cells at a multiplicity of infection of 1. Total cellular RNA was extracted at 3, 6, 9, 12, 24, 48, and 72 h after infection, and the abundance of HCV RNA and mRNA levels of the ISGs MX1, SOCS3, IFNβ, and OAS2 were quantified by qRT-PCR (Fig. 7). HCV RNA levels were undetectable or quite low until 12 h post-infection, but a substantial amount became detectable from 24 h post-infection. HCV RNA levels were comparable in KH and Huh-7.5 cells at 24 h, while its levels became significantly higher in Huh-7.5 cells compared with KH cells at 48 h. We observed a more dramatic induction of the mRNA levels of ISGs such as IFNβ and MX1 in KH cells compared with Huh-7.5 cells within 24 h when HCV RNA was low or undetectable. IFNβ mRNA levels kept increasing in KH cells after infection and peaked at 12 h; thereafter, they started to decrease. Moreover, MX1 mRNA levels kept increasing and peaked at 6 h in KH cells and then started to decrease. IFNβ and MX1 mRNA levels were continuously higher in KH cells than in Huh-7.5 cells. In contrast, the patterns of OAS2 and SOCS3 mRNA expression were a little different from those of IFNβ and MX1; we did not observe an induction of OAS2 and SOCS3 mRNA levels upon HCV infection in either KH or Huh-7.5 cells; however, OAS2 and SOCS3 mRNA levels were continuously higher in KH cells than in Huh-7.5 cells. In summary, HCV infection induced higher mRNA levels of IFNβ and MX1 in KH cells than in Huh-7.5 cells. Although HCV infection did not induce SOCS3 and OAS2 expression in either cell line, the mRNA levels of these ISGs were continuously higher in KH cells than in Huh-7.5 cells.

### Replication enhancement by IRF3 knockout and miR-122 supplementation

**Figure 8:**
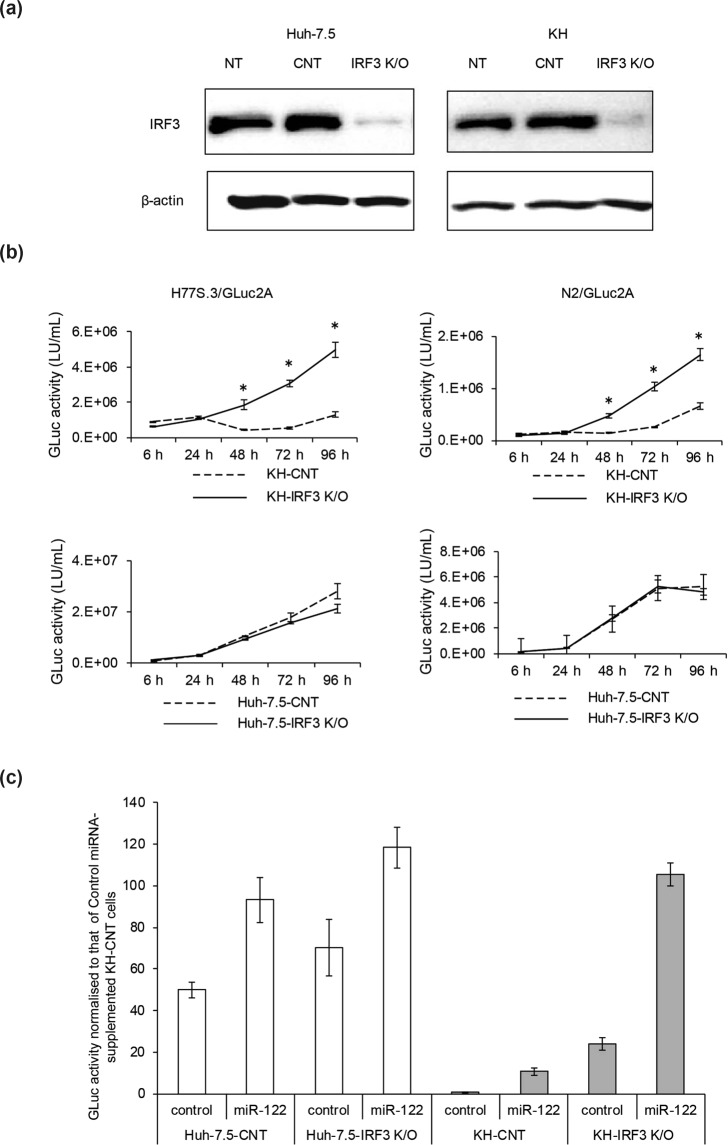
Impact of IRF3 knockout and miR-122 supplementation on HCV replication. (**a**) Efficient knockout (K/O) of IRF3 using a CRISPR-CAS9 system. Efficient K/O of IRF3 was confirmed by western blotting. Full-length gels and blots before cropping are shown in Supplementary Fig. S6. (**b**) Effect of IRF3 K/O on HCV replication. H77S.3/GLuc2A and N2/GLuc2A RNAs were transfected into KH-control (CNT) and KH-IRF3 K/O cells, as well as Huh-7.5-CNT and Huh-7.5-IRF3 K/O cells. Secreted GLuc activity was determined. Differences in the means of relative GLuc activity between CNT and IRF3 K/O cells at each time point were analysed with Student’s t test. (**c**) Effect of IRF3 K/O and miR-122 supplementation on HCV replication. Control miRNA and miR-122, were transfected into Huh-7.5/KH-CNT cells and Huh-7.5/KH-IRF3 K/O cells, and the medium was replaced at 6 h and then every 24 h. GLuc activity at 48 h after transfection was determined and normalised to that at 48 h of control miRNA-supplemented KH-CNT cells, which was set to 1.

We further examined the role of innate IFN signalling in KH cells. For this purpose, we knocked out IRF3 in both cell lines, which is a key molecule for the activation of antiviral IFN signalling and was more phosphorylated in KH cells upon HCV RNA transfection than in Huh-7.5 cells (Fig. 6d). We prepared IRF3 knockout cells by using a CRISPR-Cas9 system and confirmed efficient knockout in KH and Huh-7.5 cells by western blot analysis (Fig. 8a). We then transfected H77S.3/GLuc2A or N2/GLuc2A RNAs into IRF3 knockout KH and Huh-7.5 cells, as well as control KH and Huh-7.5 cells. While HCV replication was comparable between IRF3 knockout and control Huh-7.5 cells, it was significantly enhanced in IRF3 knockout KH cells compared with control KH cells. However, IRF3 knockout in KH cells did not increase HCV replication to a level comparable with that of Huh-7.5 cells (Fig. 8b).

As the level of miR-122 was much lower in KH cells than in Huh-7.5 cells (Fig. 2b), we examined the effect of miR-122 supplementation on HCV RNA replication and infectious virus production. Although miR-122 supplementation significantly enhanced HCV RNA replication and infectious virus production in KH and Huh-7.5 cells, the degree of enhancement was significantly greater in KH cells than in Huh-7.5 cells, indicating that miR-122 could also contribute to the inferior ability of KH cells to support HCV replication (Supplementary Fig. S2).

Next, we examined whether IRF3 knockout and miR-122 supplementation could boost HCV RNA replication level in KH cells to a similar level as in Huh-7.5 cells. However, miR-122 supplementation of control KH cells was not sufficient to boost HCV replication to the level of control Huh-7.5 cells. Finally, when IRF3 knockout KH cells were supplemented with miR-122, HCV replication was boosted to a similar level as control Huh-7.5 cells. These results indicate that the intact IFN signalling and low level of miR-122 in KH cells might restrict HCV replication (Fig. 8c).

### Antiviral activity of antiviral agents in KH cells

We compared the antiviral activity of several antiviral agents in KH and Huh-7.5 cells, including the NS3/4A inhibitor simeprevir, NS5A inhibitor daclatasvir, NS5B inhibitor sofosbuvir, and IFNα2b. We transfected HJ3-5/GLuc2A RNA into KH and Huh-7.5 cells, treated the cells with serial concentrations of the above agents, and determined the antiviral half maximal effective concentration (EC50). As shown in Supplementary Table S1, the EC50 values for the antiviral agents including DAAs and IFNα2b were comparable between both cell lines, indicating the usefulness of KH cells for antiviral screening. Although IFNα2b treatment induced the expression of two representative ISGs, OAS1 and MX1, more efficiently in KH cells than in Huh-7.5 cells (Fig. 6c), the EC50 values for IFNα2b were almost identical in KH and Huh-7.5 cells. This could be explained by the similar increase in a phosphorylated form of STAT1 upon IFNα2b treatment, which is one of the master regulators of cellular IFN responces (Supplementary Fig. S1), suggesting that some ISGs involved in the antiviral action of IFNα2b could be activated equally by IFNα2b in KH and Huh-7.5 cells.

## Discussion

HCV research and the development of antiviral medicines have been impeded due to the lack of efficient cell culture systems in which all aspects of viral lifecycle can be recapitulated. Primary human hepatocytes support the entire HCV life cycle, including cell entry, viral protein translation, viral RNA amplification, infectious virus production, and virus release to other cells. Therefore, these cells are ideal for investigating the influence of HCV on the host immune response, lipid metabolism, hepatocarcinogenesis, and other processes. However, the level of HCV replication in primary hepatocytes is low, possibly due to a robust innate antiviral response^19,20^. Therefore, several cell lines that can support HCV replication more efficiently than primary human hepatocytes have been used for basic HCV research. One of the first human hepatoma cell lines, Huh-7 cells, was reported to support efficient HCV replication^21^. Since then, several sublines of Huh-7 cells, such as Huh-7.5 and Huh-7.5.1 cells^7,22^, have been shown to support HCV replication more efficiently than the parental Huh-7 line. HCV can replicate vigorously in these sublines; thus, these lines have been used widely for screening and characterising antiviral medicines, which has resulted in the development of many highly efficient DAAs. The robust replication of HCV in Huh-7.5 cells is explained by a defect in innate immune signalling. A key molecule of innate IFN signalling, RIG-I, carries a mutation (T54I) in this cell line that results in low innate antiviral IFN responses upon HCV infection^8^.

In this study, we demonstrated that the new KH cell line, derived from human HCC, can support the entire HCV life cycle, similar to Huh-7.5 cells. KH cells could produce infectious virus, and the specific infectivity of intracellular virus was comparable between KH and Huh-7.5 cells; however, the specific infectivity of extracellular virus of KH cells was significantly lower than that of Huh-7.5 cells. These results suggest that KH cells have an inferior ability in a late step of the viral life cycle that could involve efficient viral assembly. Representative factors essential for viral entry were expressed to the same levels in KH and Huh-7.5 cells, thus demonstrating that viral entry would be permitted. The EC50 values for antiviral agents were comparable between KH and Huh-7.5 cells, showing the utility of KH cells for screening and characterising antiviral agents.

KH cells are distinct from Huh-7.5 cells in terms of the robust induction or high-level basal expression of ISGs, such as IFNβ, OAS2, MX1, and SOCS3. This can be explained by the presence of wild-type RIG-I in KH cells, in contrast to Huh-7.5 cells. KH cells retained the ability to induce ISGs on HCV RNA transfection, IFNα2b treatment, and HCV infection more robustly than Huh-7.5 cells. Phosphorylated IRF3, which represents the activation of IRF3, was more enriched upon HCV RNA transfection in KH cells than in Huh-7.5 and Huh-7 cells, and knockout of IRF3 in KH cells increased HCV replication levels, indicating that type I IFN signalling might restrict HCV replication in KH cells.

In addition to Huh-7 cells, many cell lines have been reported to support HCV replication and most of them are derived from hepatomas; however, some of these cell lines are not derived from the liver. It is difficult to predict fully whether cells can support HCV replication, but miR-122 seems to be one of the critical factors that enable its efficient replication. Although cells lacking endogenous miR-122, such as Hela and 293 cells, can support HCV replication^23^, supplementation with miR-122 is reported to make non-permissive cells permissive for HCV replication and/or infection. This phenomenon applies to non-hepatic cell lines, such as Vero cells, human embryonic kidney epithelial cells (HEK293 cells), and mouse embryotic fibroblasts, etc., as well as hepatic cell lines, such as HepG2 and Hep3B cells^24–29^. KH cells also have endogenous miR-122, but its levels were lower than in Huh-7.5 cells, which could also explain the lower levels of HCV replication in KH cells compared with Huh-7.5 cells. Supplementation with miR-122 and knockdown of IRF3, which is one of the critical factors for the innate immune response, are reported to make mouse fibroblasts permissive for HCV replication^27^. In the present study, miR-122 supplementation to KH cells augmented HCV replication; however, miR-122 supplementation was not sufficient to boost HCV replication in KH cells to the level observed in Huh-7.5 cells. Finally, both miR-122 supplementation and IRF3 knockout boosted HCV replication in KH cells to a similar level as in Huh-7.5 cells, indicating that both intact IFN signalling and the lower level of miR-122 in KH cells might restrict HCV replication. These results suggest that cells supporting HCV replication should contain the essential host factors for replication and that the balance between HCV replication and the antiviral immune response is also crucial.

KH cells are quite unique because they are derived from a Gd-EOB-DTPA uptake-positive HCC; Gd-EOB-DTPA is characterised by its rapid and specific uptake by hepatocytes via organic anion transporting polypeptides (OATPs). Therefore, Gd-EOB-DTPA uptake in the liver is thought to reflect normal hepatocyte function^30^. Between OATP1A2, 1B1, 1B3, and 2B1, only OATP1B3 expression has been found to correlate with an enhanced uptake ratio on EOB-MRI, indicating that it transports Gd-EOB-DTPA into HCC cells^31^. It is generally accepted that 85% of HCCs show hypointensity in the hepatobiliary phase of EOB-MRI compared to noncancerous liver, with a reduction of OATP1B3 protein or OATP1B3 gene expression in the tumour. In contrast, atypical Gd-EOB-DTPA uptake in the hepatobiliary phase is observed in the remaining 15% of HCCs^31,32^. A possible reason underlying the ability of KH cells to support the entire HCV life cycle could be that they maintain hepatocyte features even though they are derived from a hepatoma^9^. Several hepatoma cell lines are reported to support HCV replication, but the characteristics of hepatomas that originate from humans or other species have not been well described. For example, Huh-7 cells and Li23 cells, both of which support HCV replication, were established from human hepatomas; however, the characteristics of the original tumours, such as the absence or presence of GD-EOB-DTPA uptake, have not been well documented^33,34^. We established the KM cell line by applying the same strategies as those used to establish KH cells; KM cells were derived from a Gd-EOB-DTPA uptake-negative HCC. Interestingly, KM cells could not support HCV replication (data not shown). These results indicated that Gd-EOB-DTPA uptake of HCC could be a good predictor of whether cells established from HCC could support HCV replication.

In summary, we established a new cell line, KH cells, from Gd-EOB-DTPA uptake-positive human HCC, and KH cells can support the entire life cycle of HCV. KH cells showed higher basal levels and more robust induction of ISGs than Huh-7.5 cells, which have been the most frequently used cells in basic HCV research. This cell line will facilitate a deeper understanding of the mechanisms underlying HCV evasion of host innate antiviral responses and the establishment of persistent infection.

## Methods

### Cells

Huh-7.5 cells, a subline of the human hepatoma Huh-7 cell line^7^, and Huh-7 cells from our laboratory were grown in Dulbecco’s modified Eagle’s medium supplemented with 10% foetal bovine serum, penicillin, streptomycin, L-glutamine, and non-essential amino acids. Several hepatoma cell lines were established from primary human HCC tissues after subcutaneous transplantation into NOD/SCID mice, as described in our previous work^9^. Among the newly established cell lines, one derived from HCC tissue showing EOB uptake in dynamic MRI was used in this study, that is, KH cells. KH cells were maintained under the same conditions as Huh-7.5 cells. An A549 cell line derived from human lung carcinoma was also grown under the same conditions.

#### Sequence analysis

Total RNA was extracted from KH or Huh-7.5 cells using an RNeasy Mini Kit (QIAGEN, Hilden, Germany) according to the manufacturer’s instructions, and cDNA was synthesised with a high-capacity cDNA reverse transcription kit (Applied Biosystems, Carlsbad, CA) using random primers. For population-based sequence analysis of the N-terminal region of RIG-I, cDNA was amplified using the following set of primers: RIG-I Forward 5′-GTC CGG CCT CAT TTC CTC GGA AAA TC-3′, Reverse 5′-GGT ACA AGC GAT CCA TGA TTA TAC CCA CTA TGT TTG-3′. The amplicon was subcloned into the pGEM-T Easy Vector (Promega, Fitchburg, WI), and the N-terminus containing the amino acid at position 55 of the RIG-I sequence was determined.

#### IRF3 knockout by the CRISPR-Cas9 system

KH and Huh-7.5 cells in which IRF3 was knocked out were prepared by using a lentivirus-mediated CRISPR-CAS9 system. Briefly, the plasmid pLentiCRISPRv2 was purchased from Addgene (Watertown, MA; Plasmid#52961). The guide sequences to knock out human IRF3 were inserted into pLentiCRISPRv2, and the target guide sequence for IRF3 was AGTATTCTCCAGGGAGG. The plasmids encoding the guide sequences or an empty vector, pLentiCRISPRv2, were transfected into packaging cells (Lenti-XTM 293T cells; Takara, Shiga, Japan; #632180), with a 3rd Generation Packaging System (Applied Biological Materials, British Columbia, Canada; #LV053). The supernatants containing lentivirus were collected and used to infect KH and Huh-7.5 cells. At 48 h after infection, puromycin was added at 6 μg/mL for the transduction of lentivirus-infected cells. The puromycin-resistant cells were expanded in medium containing 6 μg/mL puromycin, and effective knockout of IRF3 was confirmed by western blot analysis. Finally, IRF3 knockout KH and Huh-7.5 cells in which IRF3 was effectively knocked out and control KH and Huh-7.5 cells, which were transfected with an empty vector, pLentiCRISPRv2, were prepared.

The other methods conducted in this study are described in Supplementary Information.

## Supplementary information

Supplementary Information

## Data Availability

All data generated or analysed during this study are included in this published article and its Supplementary Information files.
