# Supplementary material for: Establishment and Characterization of a New Cell Line Permissive for Hepatitis C Virus Infection
Source: Sci Rep. 2019 May 28;9:7943. doi: 10.1038/s41598-019-44257-5 (PMC6538753; doi:10.1038/s41598-019-44257-5)
Supplement: Supplementary file 1 — Supplementary Information [file 41598_2019_44257_MOESM1_ESM.pdf]

Supplementary Information

Establishment and Characterization of a New Cell Line Permissive for Hepatitis C Virus Infection

Hitoshi Omura[1†], Fanwei Liu[1,2†], Tetsuro Shimakami[1*], Kazuhisa Murai[1], Takayoshi Shirasaki[1], Juria Kitabayashi[1], Masaya Funaki[1], Tomoki Nishikawa[1], Ryotaro Nakai[1], Ariunaa Sumiyadorj[1], Takehiro Hayashi[1], Taro Yamashita[1], Masao Honda[1], and Shuichi Kaneko[1]

[1] Department of Gastroenterology, Kanazawa University Graduate School of Medicine, Kanazawa, Ishikawa 920-8641, Japan

[2] State Key Laboratory for Diagnosis and Treatment of Infectious Diseases, Collaborative Innovation Center for Diagnosis and Treatment of Infectious Diseases, The First Affiliated Hospital, College of Medicine, Zhejiang University, Hangzhou, 310003, China

[†]Both authors contributed equally to this work

[*]To whom correspondence should be addressed: Tetsuro Shimakami, MD
Department of Gastroenterology, Kanazawa University Hospital,
13-1 Takaramachi, Kanazawa, Ishikawa 920-8641, Japan
Tel: 81-76-265-2233; Fax: 81-76-234-4250; E-mail: shimakami@m-kanazawa.jp

A list of the information included

Supplementary Table S1
Supplementary Figure S1
Supplementary Figure S2
Supplementary Figure S3
Supplementary Figure S4
Supplementary Figure S5
Supplementary Figure S6
Supplementary Figure S7
Supplementary Methods
References for Supplementary Information

Supplementary Table S1 EC50 of antiviral agents for the HJ3-5 virus

	Simeprevir (nM)	Daclatasvir (pM)	Sofosbuvir (nM)	IFNα2b (IU/mL)
KH	12.1	62.3	170.7	10.7
Huh-7.5	12.4	58.8	174.6	8.7

Supplementary Figure S1

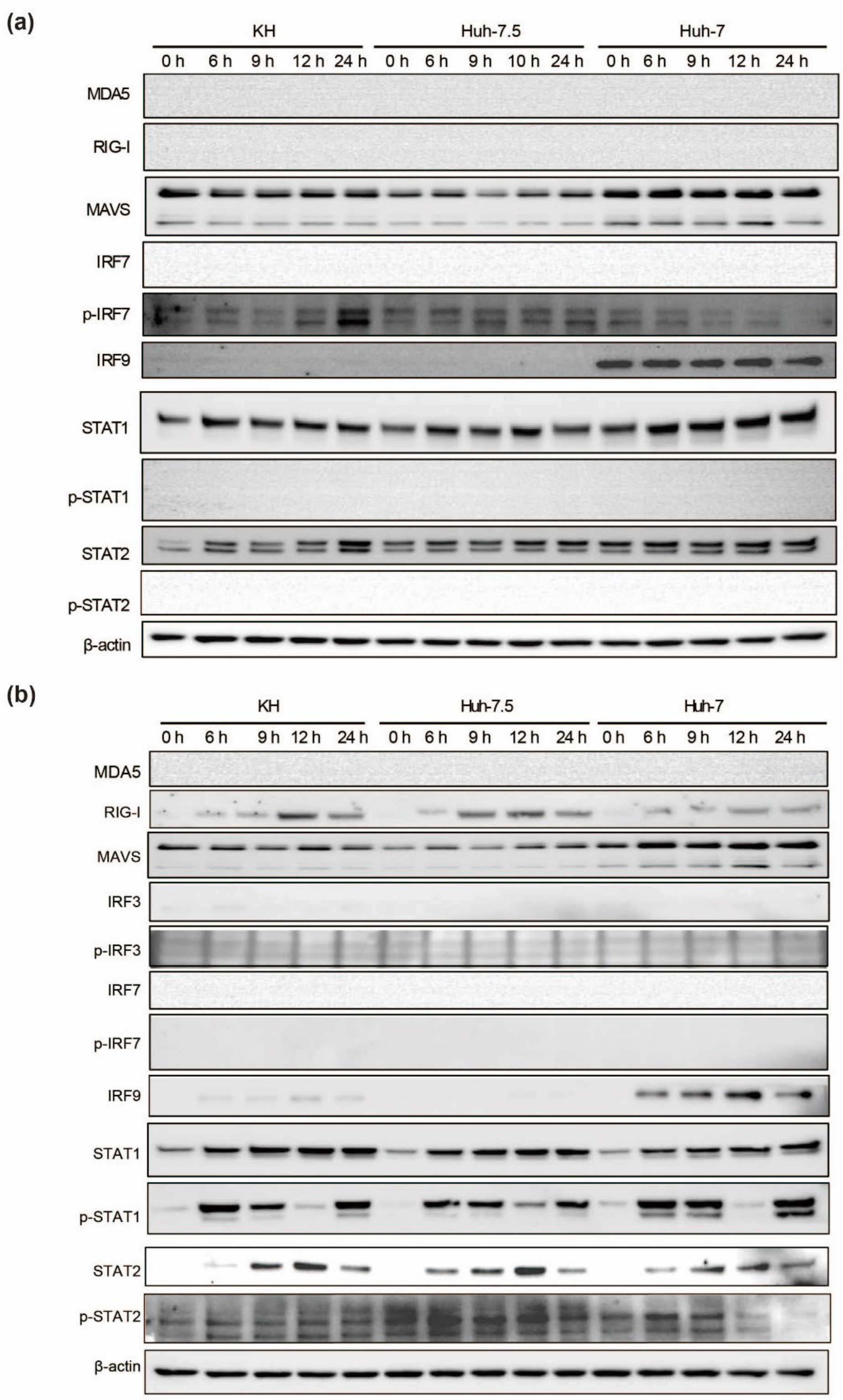

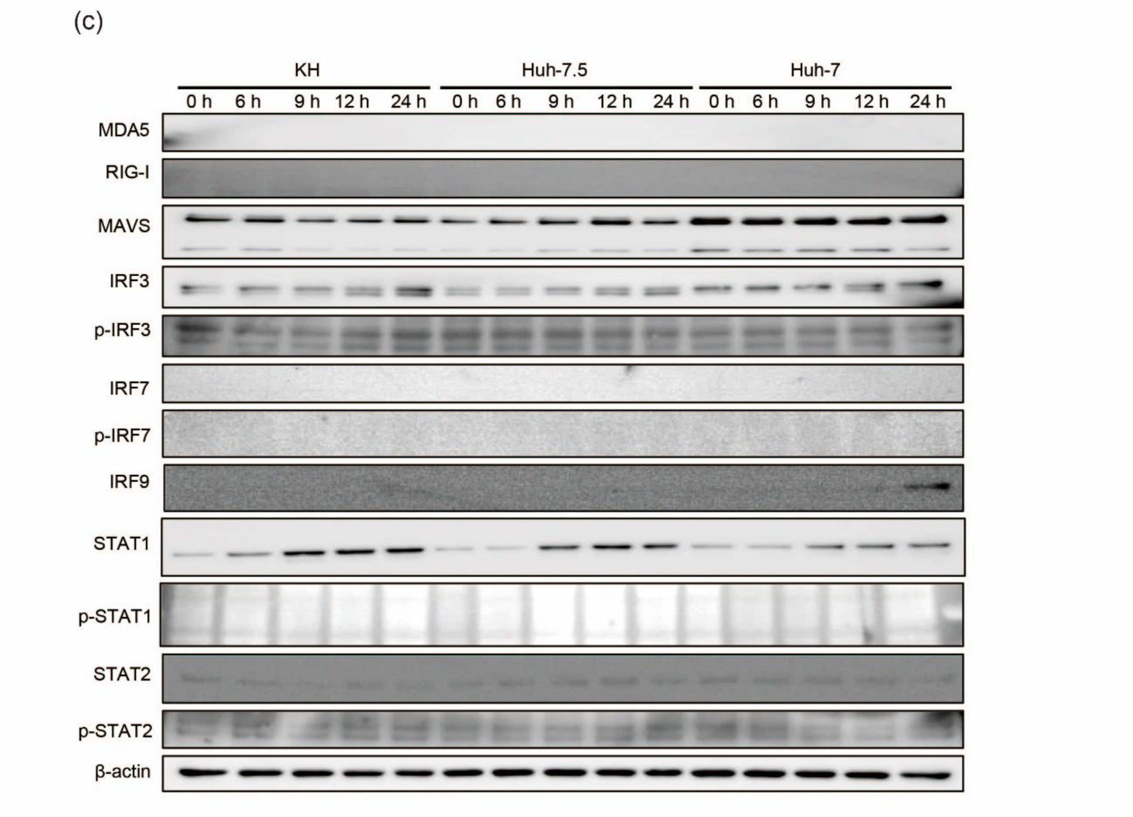

Supplementary Figure S1. Impact of HCV RNA transfection, IFN treatment, and poly(I:C) transfection on ISG expression. HCV RNA coding translation-incompetent JFH1 A338U and poly(I:C) were transfected into KH, Huh-7.5, and Huh-7 cells. IFNα2b was added to these cells. Then, at 0, 6, 9, 12, and 24 h after transfection or IFNα2b addition, western blot analyses were performed by using appropriate antibodies. (a) HCV RNA transfection. (b) IFNα2b addition. (c) Poly(I:C) transfection. Full-length gels and blots before cropping are shown in Supplementary Figure S7.

Supplementary Figure S2

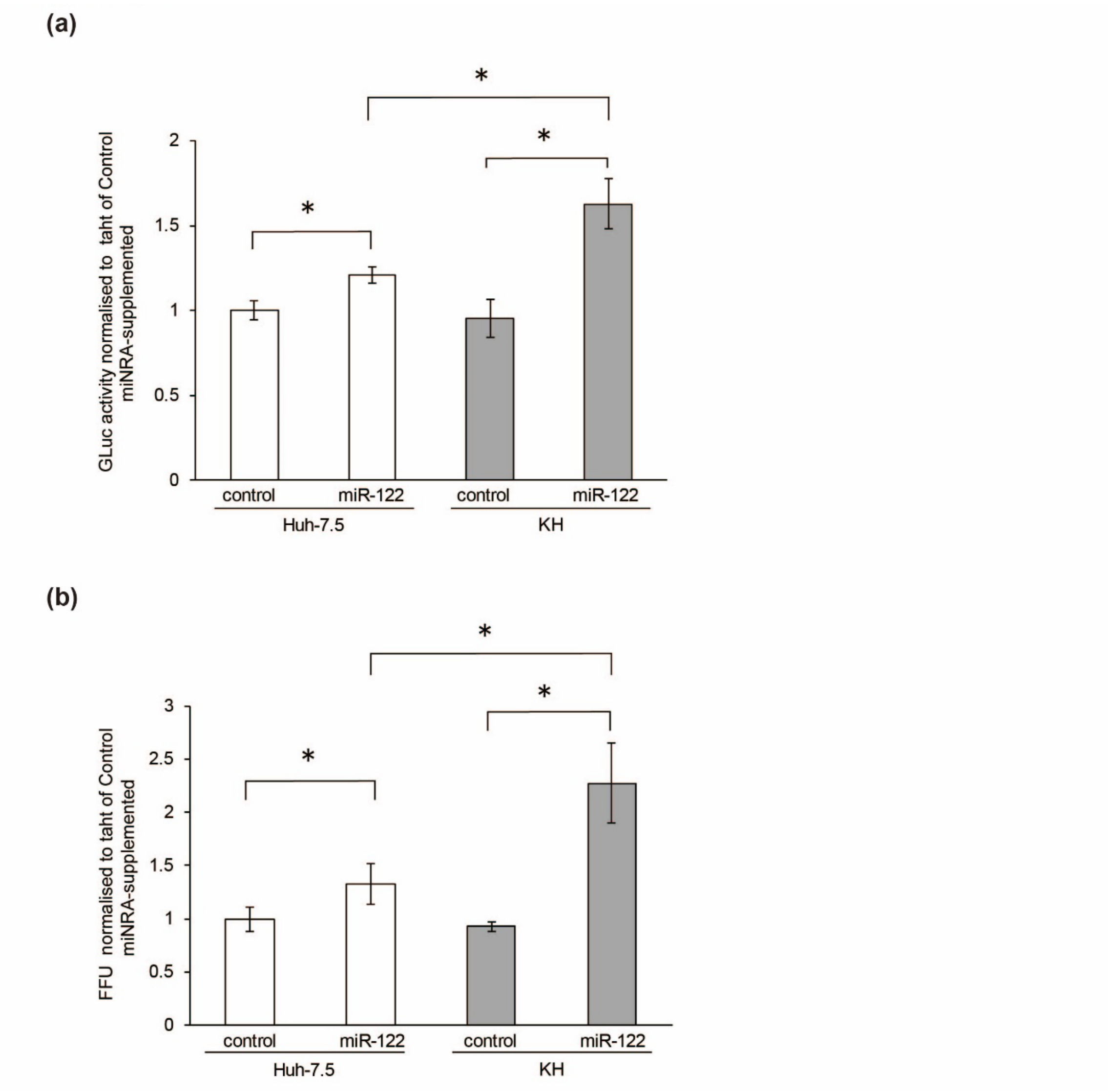

Supplementary Figure S2 Impact of miR-122 supplementation on HCV replication and infectious virus production. miR-122 or control miRNA was transfected into KH or Huh-7.5 cells, and 24 h later, the cells were transfected with HJ3-5/GLuc2A RNA. Then, 6 h later, the medium was replaced with fresh medium and collected for normalization of HCV RNA transfection. Subsequently, the medium was replaced with fresh medium and collected for GLuc or FFU assay every 24 h until 48 h after HCV RNA transfection. (a) Impact on HCV replication. GLuc activity at 48 h was determined and normalised to GLuc activity at 6 h, and further normalised to GLuc activity from control miRNA-supplemented cells, which was set to 1 in each cell line. (b) Impact on infectious virus production. The medium at 48 h was used to infect naïve Huh-7.5 cells, and at 72 h after infection, we performed an FFU assay to determine infectious virus yield. FFUs from miR-122-supplemented cells were normalised to that from control miRNA-supplemented cells, which was set to 1 in each cell line. Differences between the shown combinations in (a) and (b) were analyzed by Student’s t test.

Supplementary Figure S3

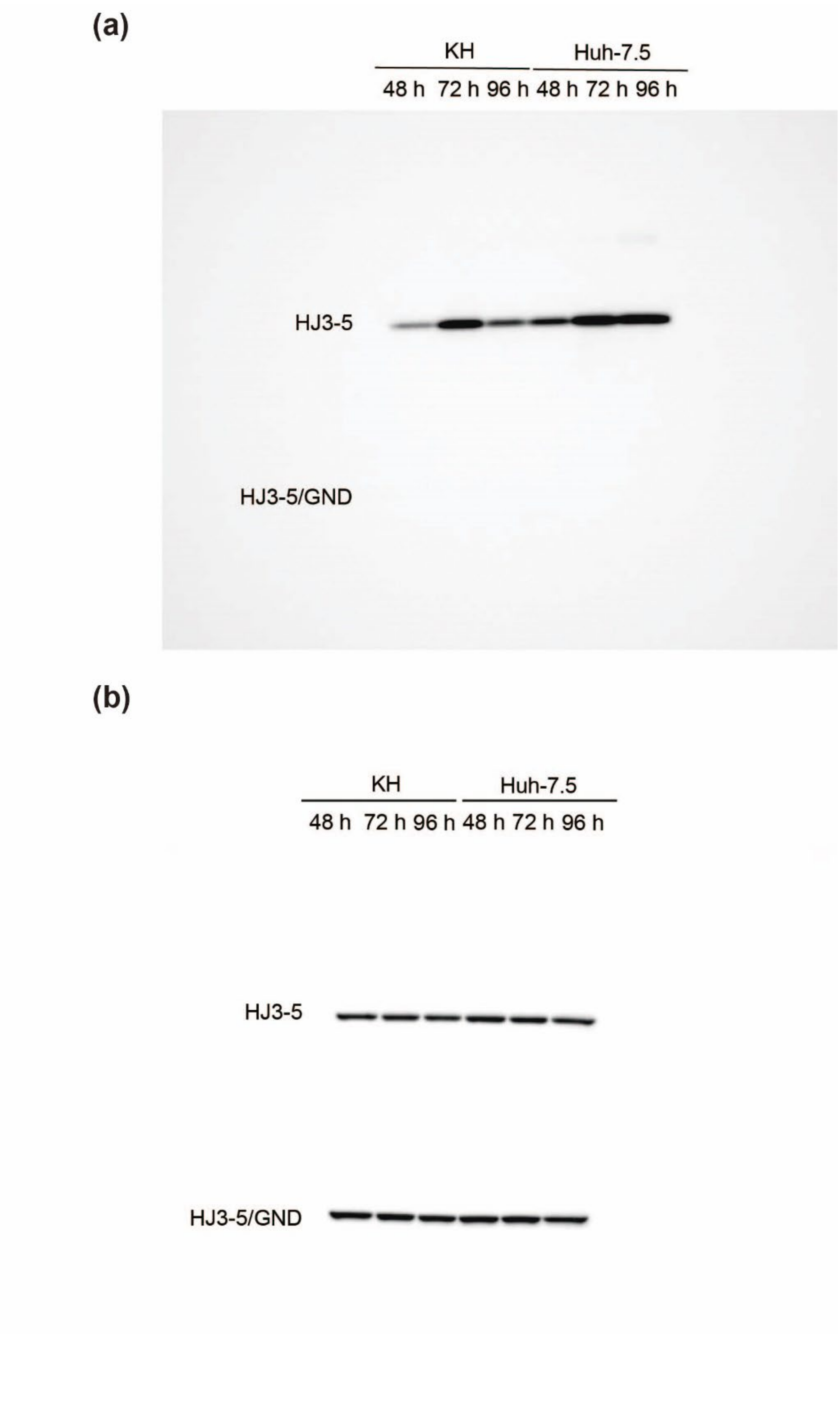

Supplementary Figure S3. Full-length gels and blots before cropping for Figure 3a.
(a) Anti-HCV core antibody. (b) Anti-β-actin antibody.

Supplementary Figure S4

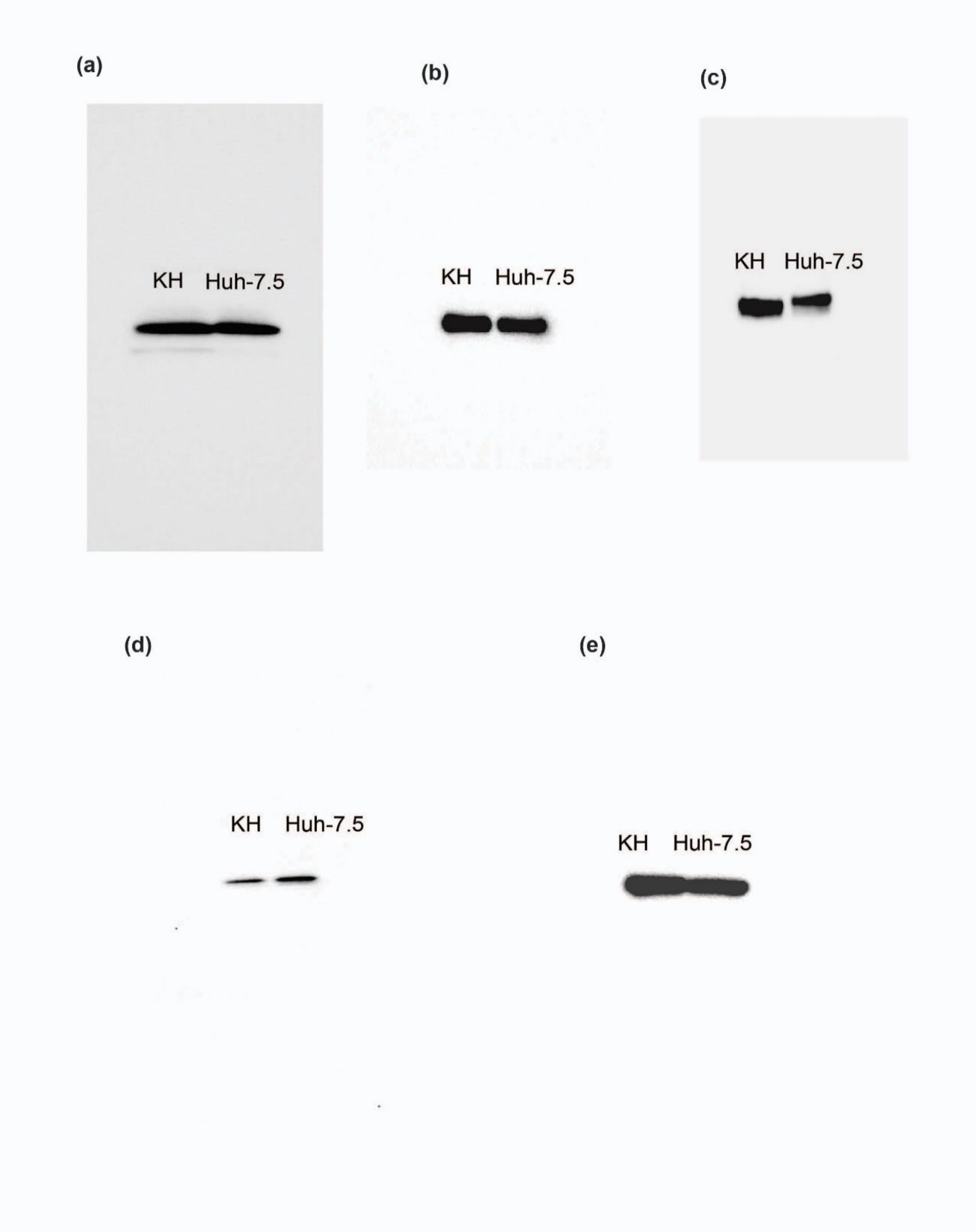

Supplementary Figure S4. Full-length gels and blots before cropping for Figure 5c. (a) Anti-claudin1 antibody. (b) Anti-occludin antibody. (c) Anti-SR-B1 antibody. (d) Anti-CD81 antibody. (e) Anti-β-actin antibody.

Supplementary Figure S5

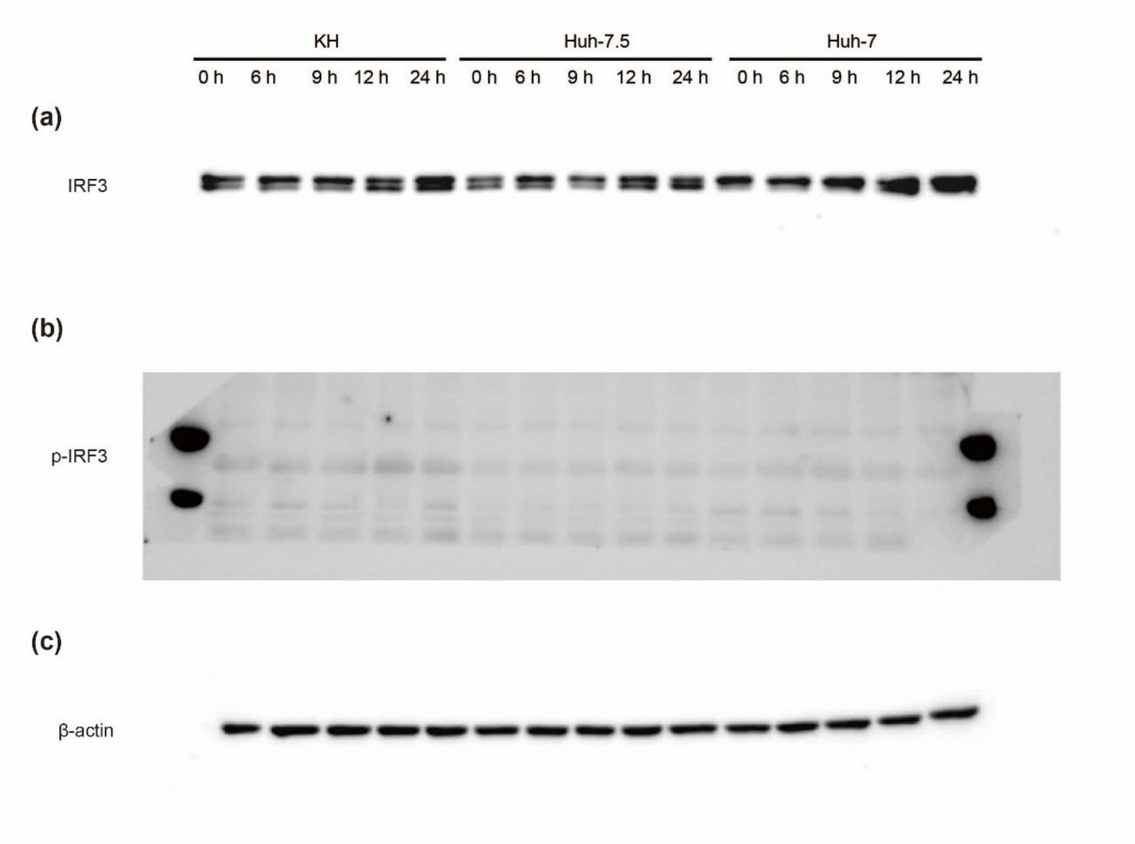

Supplementary Figure S5. Full-length gels and blots before cropping for Figure 6d. (a) Anti-IRF3 antibody. (b) Anti-p-IRF3 antibody. (c) Anti-β-actin antibody.

Supplementary Figure S6

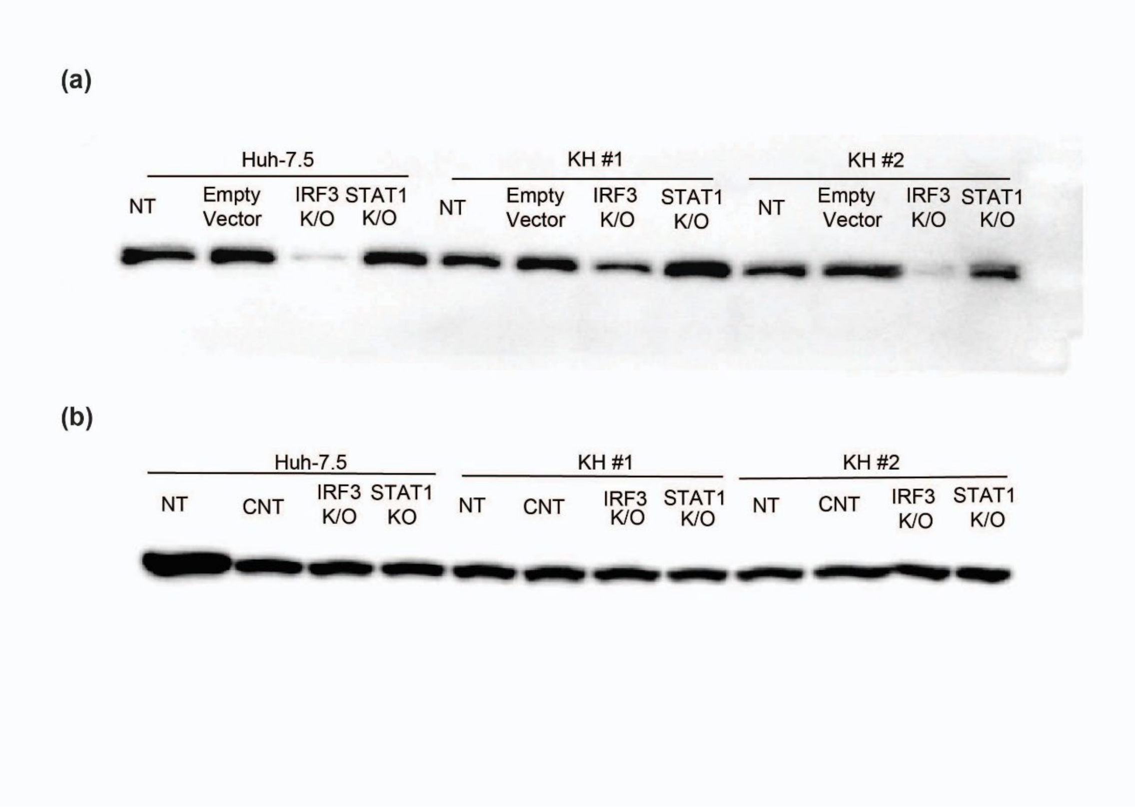

Supplementary Figure S6. Full-length gels and blots before cropping for Figure 8a. (a) Anti-IRF3 antibody. (b) Anti-β-actin antibody. KH #1 and #2 cells were selected by puromycin in separate experiments. We used KH #2 cells because IRF3 was knocked down more efficiently in these cells than in KH #1 cells. STAT1 KO cells were prepared for a different purpose.

Supplementary Figure S7

(a)

MDA5

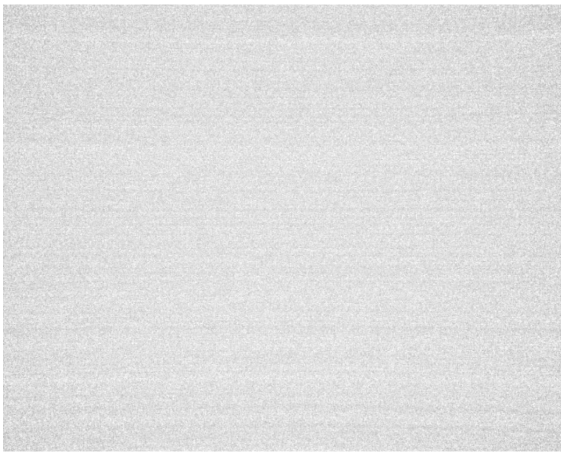

IRF7

RIG-I

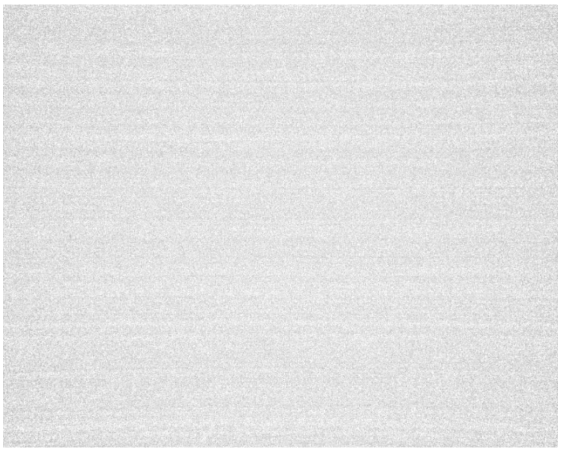

p-IRF7

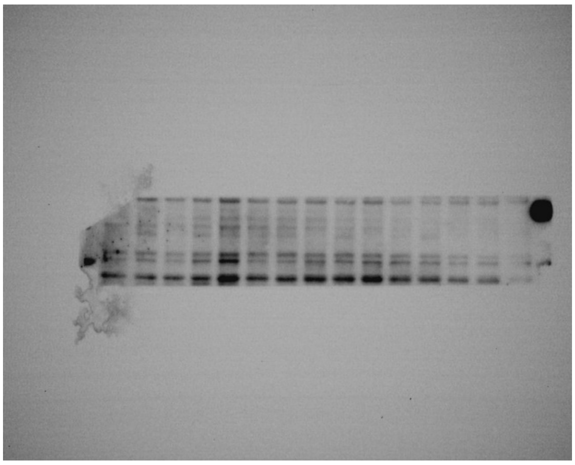

MAVS

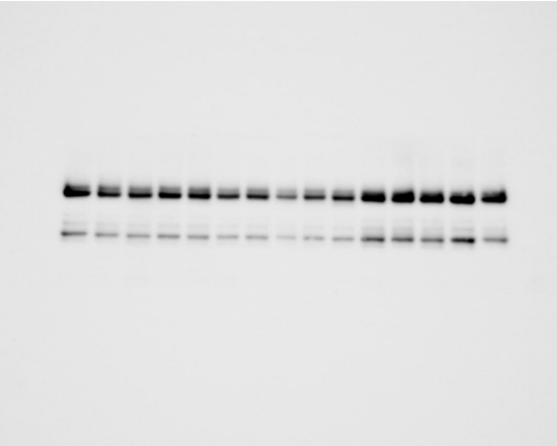

IRF9

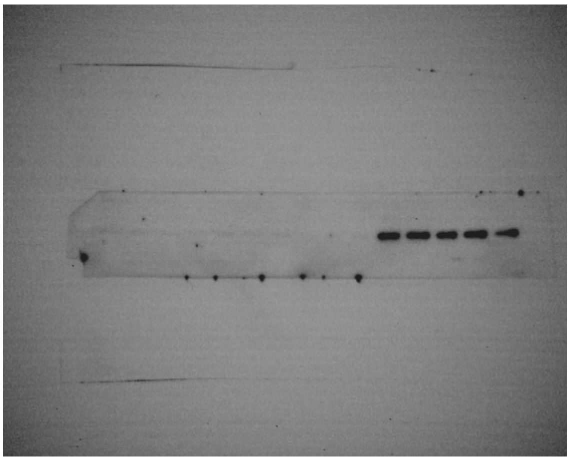

STAT1

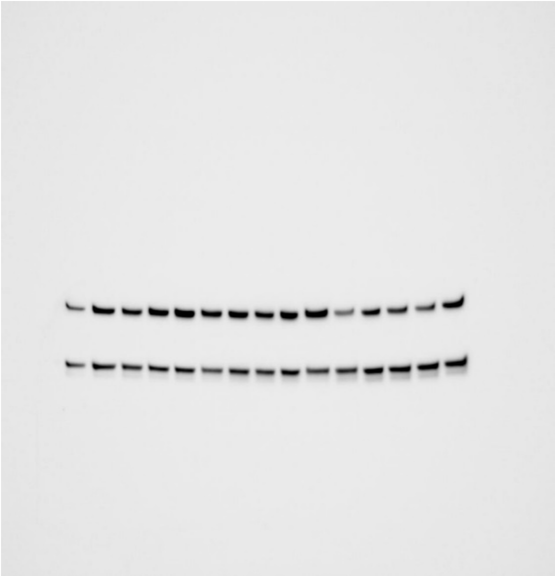

p-STAT1

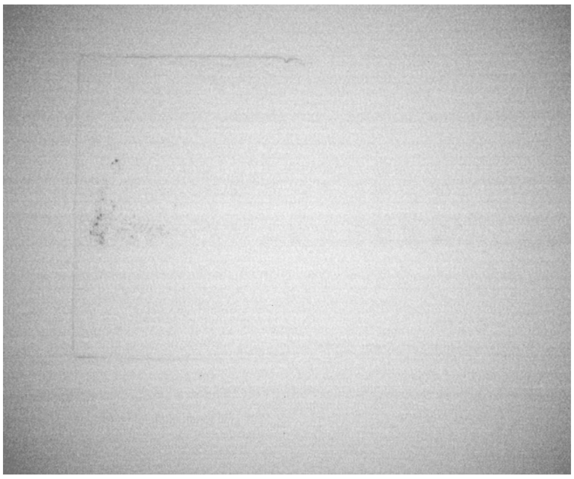

STAT2

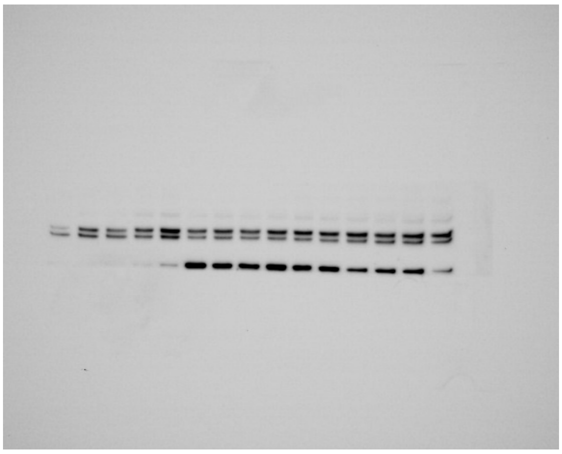

p-STAT2

β-actin

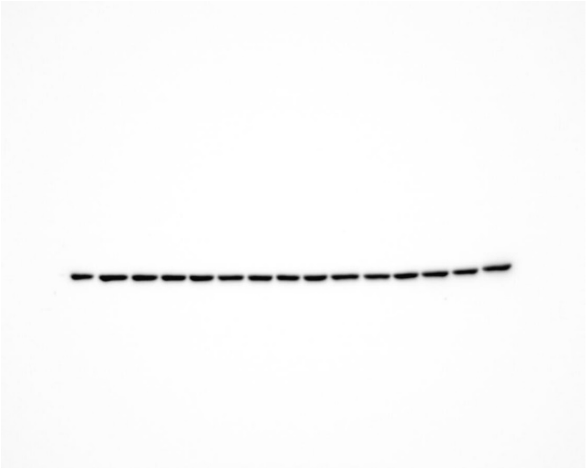

(b)

MDA5

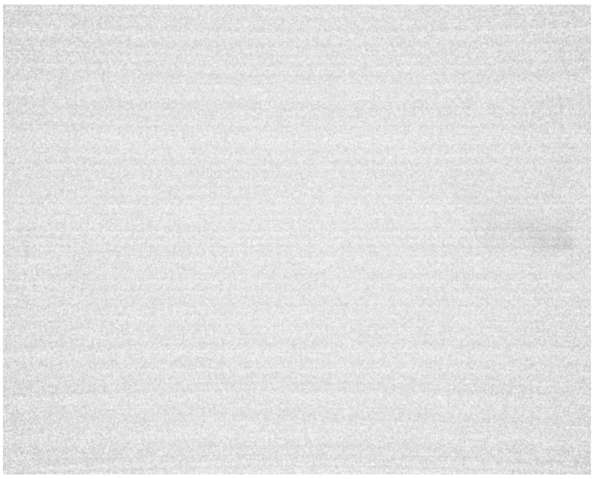

RIG-I

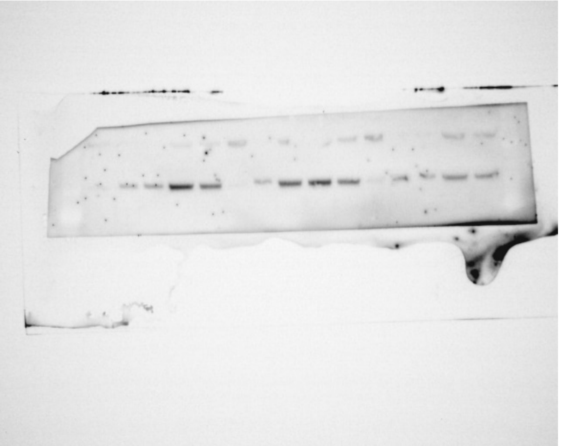

p-IRF3

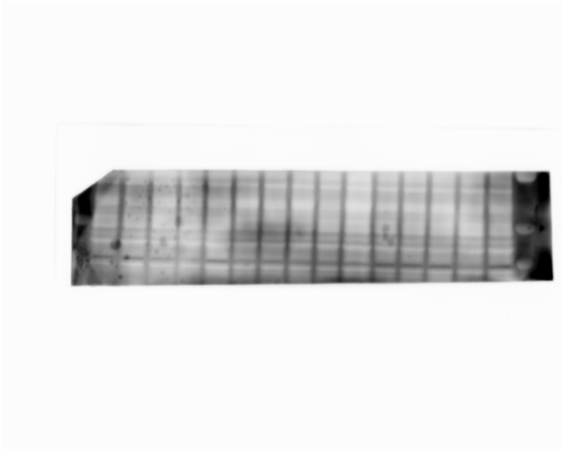

MAVS

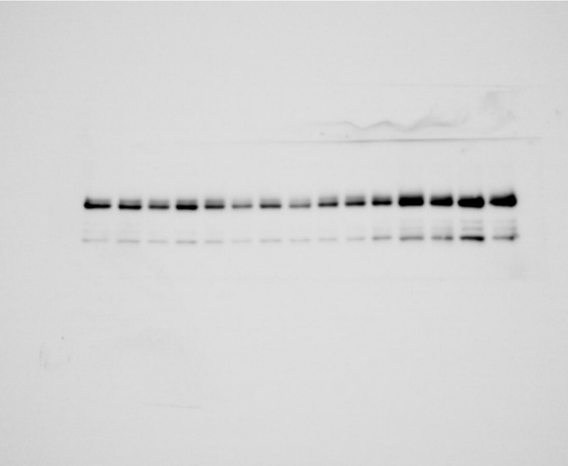

IRF7

IRF3

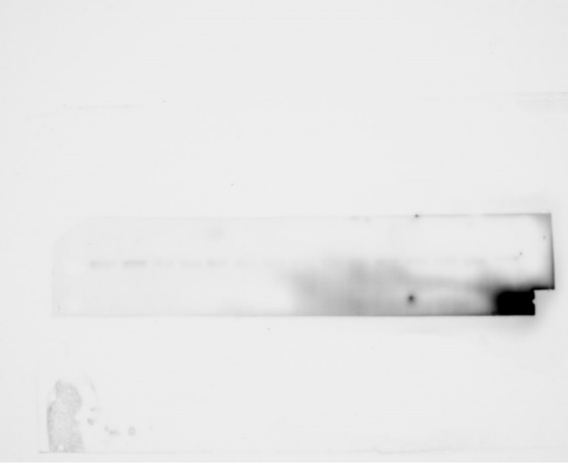

p-IRF7

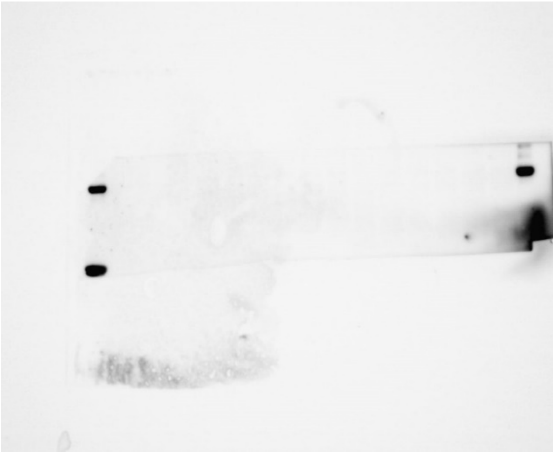

IRF9

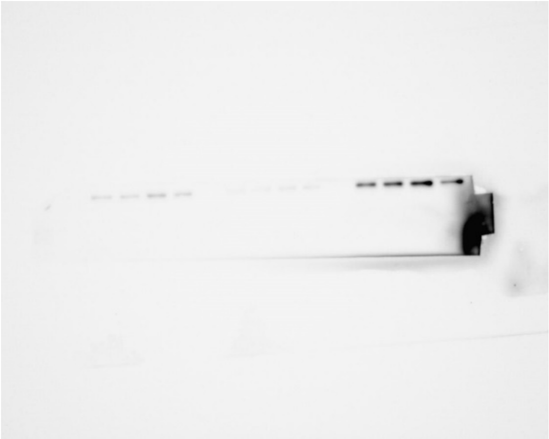

STAT2

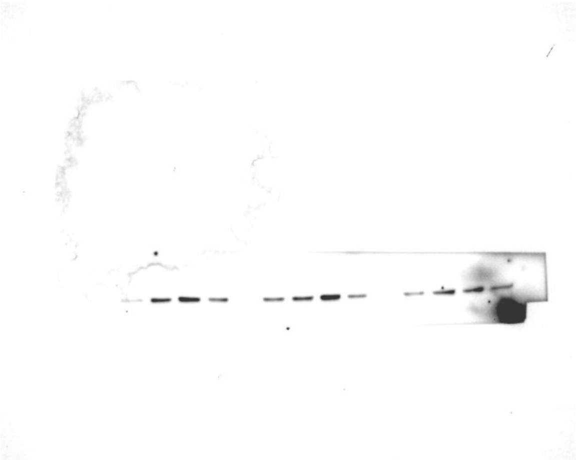

STAT1

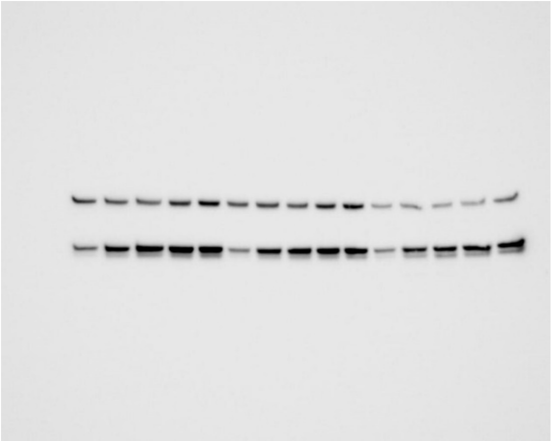

p-STAT2

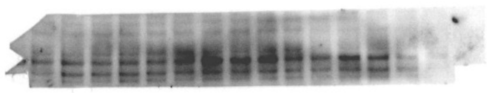

p-STAT1

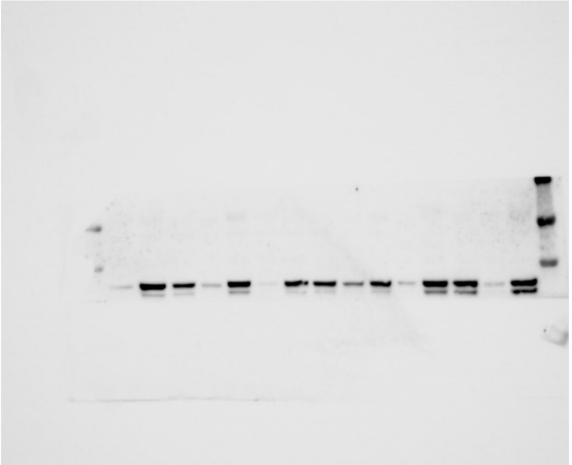

β-actin

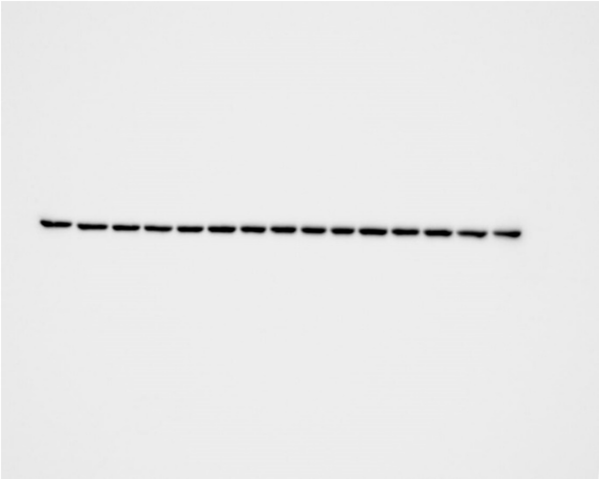

(c)

MDA5

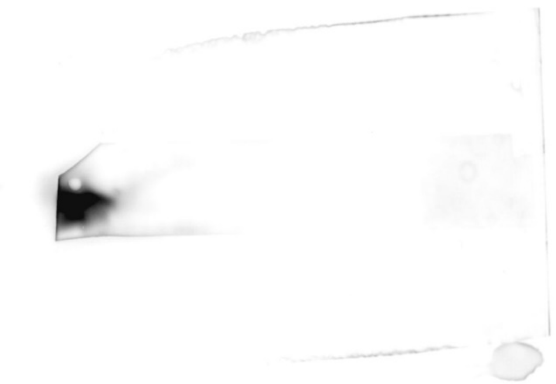

RIG-I

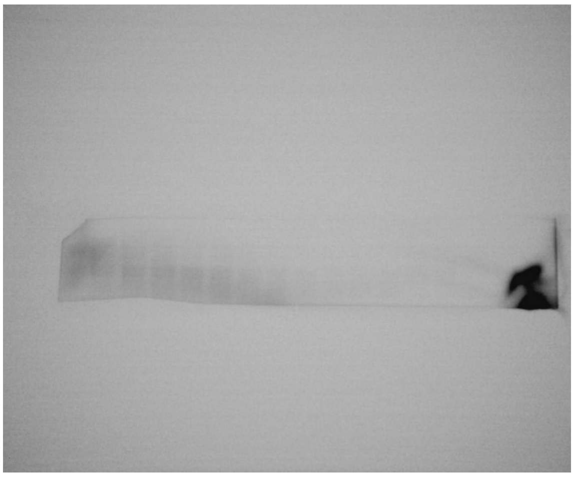

MAVS

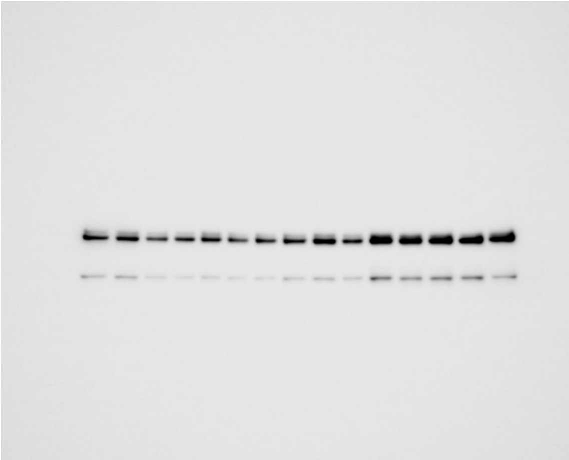

IRF3

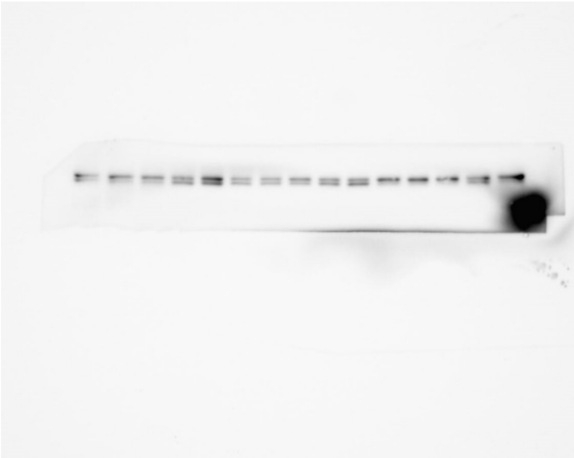

p-IRF3

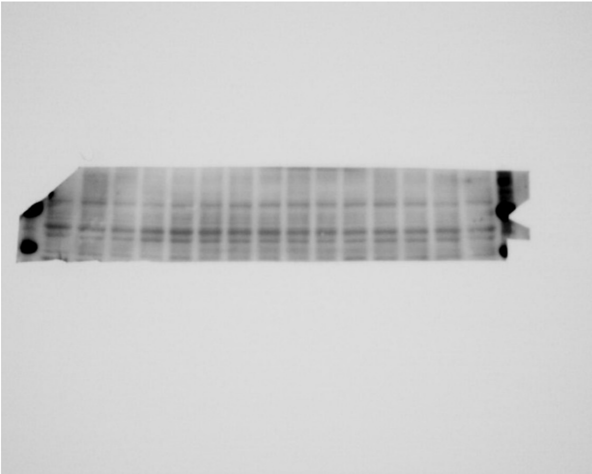

IRF7

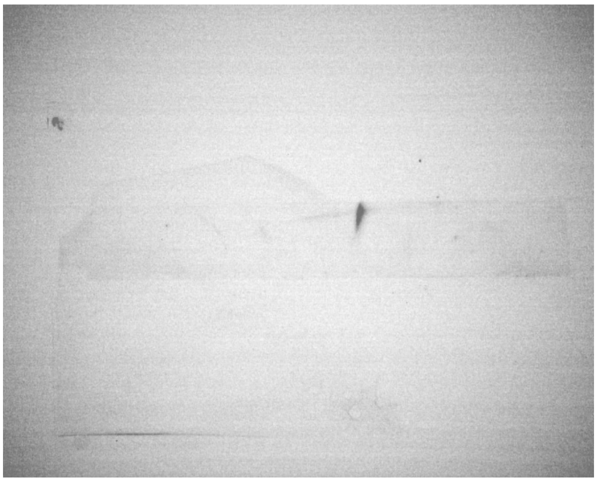

p-IRF7

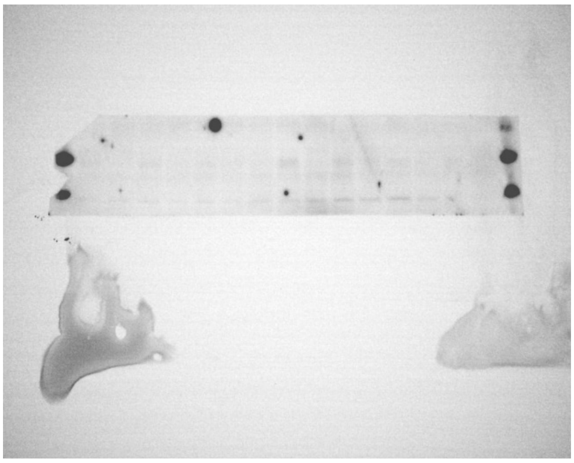

p-STAT1

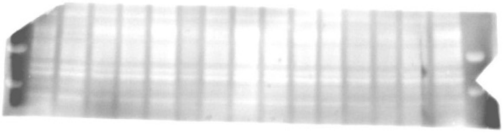

IRF9

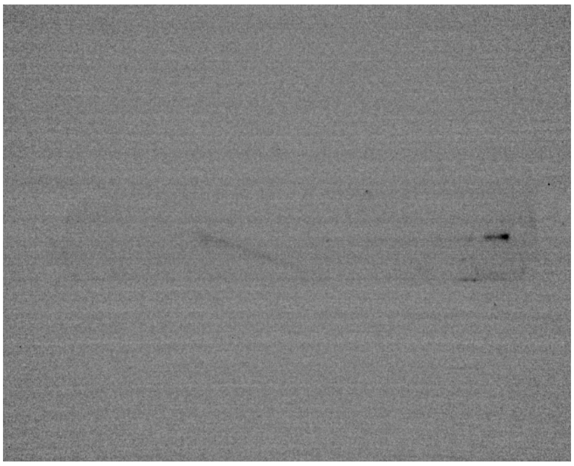

STAT2

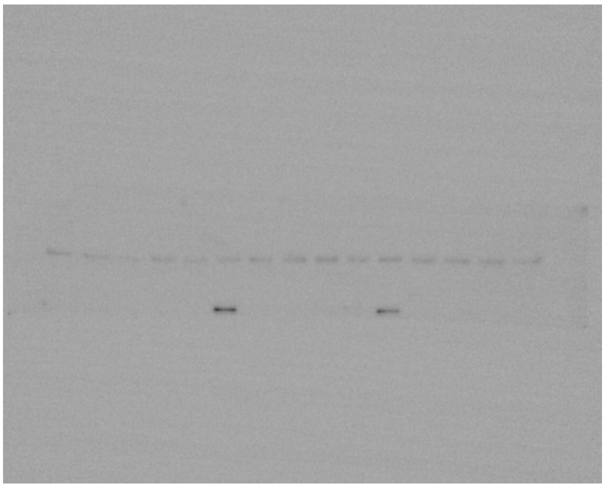

STAT1

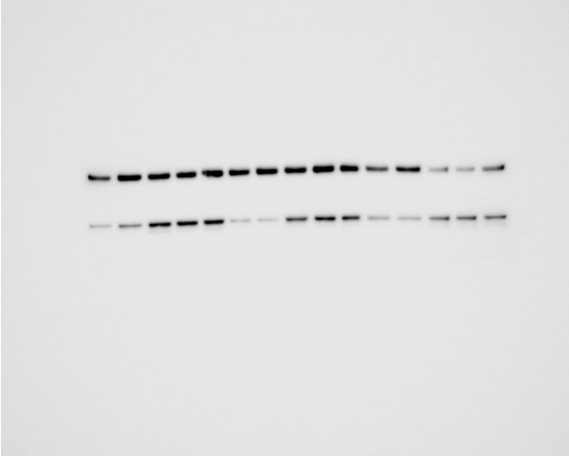

p-STAT2

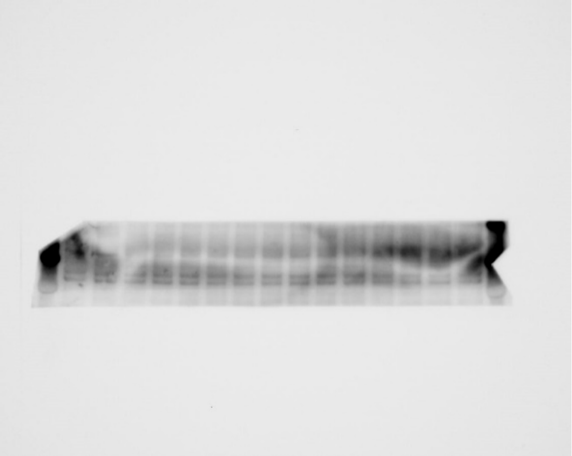

β-actin

Supplementary Figure S7. Full-length gels and blots before cropping for Supplementary Figure S1. (a) These correspond to Supplementary Figure 1a. (b) These correspond to Supplementary Figure 1b. (c) These correspond to Supplementary Figure 1c.

Supplementary Methods

Plasmids

The plasmid pH77S.3[1] is derived from pH77S,[2] an infectious molecular clone of genotype 1a HCV that contains several adaptive mutations. The *Gaussia* luciferase (GLuc) coding sequence, fused at its C terminus to the foot-and-mouth disease virus 2A autoprotease, was inserted between p7 and NS2 of pH77S.3 and designated pH77S.3/GLuc2A.[1] The chimeric infectious molecular clone pHJ3-5 is comprised of the structural protein-coding region of the H77c virus placed within the background of the genotype 2a JFH1 virus.[2] The plasmids pN2/GLuc2A,[3] pJFH1/GLuc2A,[3] and pHJ3-5/GLuc2A[4] encoding genotype 1b N,[5] genotype 2a JFH1,[6] and genotype 1a/2a chimera HJ3-5 with the GLuc-coding sequence, respectively, were used. pJFU1 A338U[7] coding a translation-incompetent HCV internal ribosome entry site mutant was used.

Reagents

Simeprevir and daclatasvir were purchased from Funakoshi Co., Ltd. (Tokyo, Japan) and sofosbuvir was obtained from Cayman Chemical (Ann Arbor, MI). Each was prepared in dimethyl sulfoxide (DMSO). All final dilutions contained 0.5% DMSO.

Metaphase chromosome preparation

Metaphase chromosome spreads from human cells were prepared according to standard procedures. Briefly, the cells were cultured, treated with colcemid (0.02 µg/mL) for 2 h, and harvested. After treatment with 0.075 M KCl for 20 min at room temperature, the cells were fixed 3 times with MeOH:acetic acid (3:1) and the fixed cells were spread on slides.

Multicolor-FISH (M-FISH) analysis

M-FISH was performed using commercially available kits (MetaSystems, Inc., Altlussheim, Germany) according to the manufacturer's protocol with some modifications. Briefly, metaphase spreads were hardened overnight at 70 °C. After applying M-FISH probes to the metaphase spreads, co-denaturation of target DNA with probe DNA was performed for 5 min at 68 °C, followed by incubation for 72 h at 37 °C to allow probe hybridization. The slides were washed with 50% formamide/2× standard saline citrate (SSC) solution for 20 min at 37 °C and 1× SSC for 5 min at room temperature. The slides were counterstained with 4′,6-diamidino-2-phenylindole and mounted. Separate fluorochrome images were captured using a Leica DC 350FX cooled CCD camera (Leica Microsystems, Inc., Buffalo Grove, IL) mounted on a Leica DMRA2 microscope using Leica CW4000 FISH software. The images were analyzed using Leica

CW4000 Karyo (Leica).

In vitro transcription and RNA transfection

RNA was synthesized with the T7 RiboMAX™ Express Large-Scale RNA Production System (Thermo Fisher Scientific, Inc., Waltham, MA) after linearization of the plasmids with *Xba*I. Following treatment with RNase-free DNase to remove the template DNA, RNA was purified using an RNeasy Mini Kit (QIAGEN). RNA transfection was carried out using a TransIT mRNA Transfection Kit (Takara, Shiga, Japan), according to the manufacturer's protocol or electroporation as described previously.[4]

miRNA transfection

miR-122 (MISSION micro RNA mimic, hsa-miR-122, HMI1002) and control miRNA (MISSION miRNA, Negative Control 1, HMC0002) were purchased from Sigma-Aldrich (St. Louis, MO) and reconstituted in water. Each miRNA was transfected at 20 nM by using Lipofectamine 2000 (Thermo Fisher Scientific, Inc.).

Luciferase activity assay

Following RNA transfection, cell culture supernatants were collected, and fresh medium was added at 24-h intervals. Secreted GLuc activity was measured in 50-µL aliquots of the supernatant using a GLuc Assay Kit (New England BioLabs, Ipswich, MA) according to the manufacturer's protocol. Luminescent signals were measured on a GloMax®-Multi+ Microplate Multimode Reader with Instinct® (Promega).

qRT-PCR of HCV RNA and mRNAs of host proteins

Total RNA was isolated using an RNeasy Mini Kit (QIAGEN), and cDNA was synthesized with a High-Capacity cDNA Reverse Transcription Kit (Applied Biosystems). The primer pairs and probes for the following genes, CYP3A4, ApoA1, albumin, AFP, HNF4α, OATP1B3, CK19, EpCAM, and β-actin, were obtained from the TaqMan assay reagents library. HCV RNA was quantified as described previously.[8]

Western blotting and immunostaining analyses

Western blotting and immunostaining were performed as described previously.[7,9] The expression of HCV core protein, CD81, SR-B1, occludin, claudin-1, IRF3, p-IRF3, STAT1, p-STAT1, STAT2, p-STAT2, IRF7, p-IRF7, IRF9, MAVS, RIG-I, MDA5, and β-actin was evaluated with the following antibodies: mouse monoclonal anti-HCV core C7-50 (#MA1-080; Thermo Fisher), rabbit monoclonal anti-CD81 (#109201; Abcam Cambridge, MA), anti-SRB1 (#52629; Abcam), rabbit monoclonal anti-occludin (#167161; Abcam), rabbit polyclonal anti-claudin1 (#15098; Abcam), rabbit monoclonal anti-IRF3 (#4302; Cell Signaling Technology, Danvers, MA), rabbit monoclonal anti-p-IRF3 (#29047, Cell Signaling Technology), rabbit polyclonal anti-STAT1 (#9172; Cell Signaling Technology), rabbit monoclonal anti-p-STAT1 (#7649; Cell Signaling Technology), rabbit polyclonal anti-STAT2 (#4594; Cell Signaling Technology), rabbit polyclonal anti-p-STAT2 (#4441; Cell Signaling Technology), rabbit polyclonal anti-IRF7 (#4920; Cell Signaling Technology), rabbit polyclonal anti-phosphorylated IRF7 (#5184; Cell Signaling Technology), rabbit polyclonal anti-IRF9 (#4920; Cell Signaling Technology), rabbit monoclonal anti-RIG-I (#3743; Cell Signaling Technology), rabbit polyclonal anti-MAVS (#3993; Cell Signaling Technology), rabbit polyclonal anti-MDA5 (#5321; Cell Signaling Technology), and rabbit polyclonal anti-β-actin (#4967; Cell

Signaling Technology).

Immunofluorescence analysis

Immunofluorescence analysis was performed as described previously.[10]

Focus-forming unit (FFU) assay

An FFU assay was performed as described previously.[1,10]

Quantitation of western blots

Signal strength of HCV core protein in western blots was quantified using ImageJ software developed at the National Institutes of Health.

miR-122 quantification

Total RNA containing miRNAs was isolated according to the protocol of the mirVana miRNA Isolation Kit (Thermo Fisher Scientific, Inc.). The abundance of miR-122 and RNU6B was determined by a quantitative PCR method as described in our previous study.[9]

IFN treatment and HCV RNA and poly(I:C) transfection

IFNα2b and low molecular weight poly(I:C) were purchased from Sigma-Aldrich and reconstituted in water. To examine the effects of IFNα2b treatment, HCV RNA, and poly(I:C) transfection on ISG induction and expression, KH, Huh-7.5, and Huh-7 cells were seeded at 5.0×10^5 cells/well in a 6-well plate. After 12 h, IFNα2b was added at various concentrations, HCV RNA coding translation-incompetent JFH1 A338U and

poly(I:C) were transfected by using Lipofectamine 3000 (Thermo Fisher) at 2 and 10 μg/well, respectively.

Antiviral activity assay

The indicated HCV RNAs were transfected by electroporation. The medium was replaced with fresh medium containing serial dilutions of the antiviral compounds at 48 h after transfection and at 24 h intervals thereafter. Secreted GLuc activity was determined at 72 h after adding the antiviral compounds. The concentration of each compound required to reduce the amount of secreted GLuc activity by 50% (EC50) was determined using a 3-parameter Hill equation (Sigma Plot 10.0).

Intra- and extra-cellular infectivity assay and specific infectivity of intra- and extra-cellular virus

HJ3-5 RNA was transfected by electroporation into KH and Huh-7.5 cells, and after 72 h, cell pellets harvested after trypsinization were resuspended in complete medium, washed twice with phosphate-buffered saline, and lysed by 4 cycles of freezing and thawing. The lysates were clarified by centrifugation at 2,300 × *g* for 5 min prior to inoculation onto naïve Huh-7.5 cells. At the same time, extra-cellular medium was also collected. The media derived from extra- and intra-cellular cultures were used to infect naïve Huh-7.5 cells, which were plated in 48-well plates at a density of 4.0×10^4 cells/well at 24 h prior to infection. The amount of HCV RNA in intra- and extra-cellular media was quantitated by qRT-PCR to determine specific infectivity. At 72 h after infection, we performed an FFU assay to determine infectious virus yield in cells cultured in intra- and extra-cellular media. The secretion ratio of virus from intra- to extra-cellular cells was

calculated from the intra-and extra-cellular infectious virus yield.

Statistical analysis

The results are expressed as the mean ± standard deviation. Data were analyzed with Student's t test and one-way analysis of variance with Tukey's multiple comparisons test and considered statistically significant at $p < 0.05$; * represents $p < 0.05$. Statistical analysis was performed using GraphPad Prism7 (GraphPad Software, Inc., La Jolla, CA).

References for Supplementary Information

1 Shimakami, T. *et al.* Protease inhibitor-resistant hepatitis C virus mutants with reduced fitness from impaired production of infectious virus. *Gastroenterology* **140**, 667–675; 10.1053/j.gastro.2010.10.056 (2011).

2 Yi, M., Ma, Y., Yates, J. & Lemon, S. M. Compensatory mutations in E1, p7, NS2, and NS3 enhance yields of cell culture-infectious intergenotypic chimeric hepatitis C virus. *J. Virol.* **81**, 629–638; 10.1128/JVI.01890-06 (2007).

3 Yamane, D. *et al.* Regulation of the hepatitis C virus RNA replicase by endogenous lipid peroxidation. *Nat. Med.* **20**, 927–935; 10.1038/nm.3610 (2014).

4 Shimakami, T. *et al.* Stabilization of hepatitis C virus RNA by an Ago2-miR-122 complex. *Proc. Nat. Acad. Sci. U. S. A.* **109**, 941–946; 10.1073/pnas.1112263109 (2012).

5 Beard, M. R. *et al.* An infectious molecular clone of a Japanese genotype 1b hepatitis C virus. *Hepatology* **30**, 316–324; 10.1002/hep.510300137 (1999).

6 Wakita, T. *et al.* Production of infectious hepatitis C virus in tissue culture from a cloned viral genome. *Nat. Med.* **11**, 791–796; 10.1038/nm1268 (2005).

7 Shirasaki, T. *et al.* La protein required for internal ribosome entry site-directed translation is a potential therapeutic target for hepatitis C virus replication. *J. Infect. Dis.* **202**, 75–85; 10.1086/653081 (2010).

8 Honda, M., Shimazaki, T. & Kaneko, S. La protein is a potent regulator of replication of hepatitis C virus in patients with chronic hepatitis C through internal ribosomal entry site-directed translation. *Gastroenterology* **128**, 449–462 (2005).

9 Shirasaki, T. *et al.* MicroRNA-27a regulates lipid metabolism and inhibits hepatitis C virus replication in human hepatoma cells. *J. Virol.* **87**, 5270–5286; 10.1128/JVI.03022-12 (2013).

10 Shimakami, T. *et al.* The acyclic retinoid Peretinoin inhibits hepatitis C virus replication and infectious virus release in vitro. *Sci. Rep.* **4**, 4688; 10.1038/srep04688 (2014).
